# Systematic Review of Vancomycin Population Pharmacokinetic Models in Critically Ill Patients: A Focus on Model Characteristics and External Predictive Performance

**DOI:** 10.3390/antibiotics15100948

**Published:** 2026-09-24

**Authors:** Tianyu Xu, Hao Sun, Yujia Zhang, Ying Zhang, Limei Zhao, Chengbin Wang, Mingming Zhao

**Affiliations:** 1 Department of Pharmacy, Shengjing Hospital of China Medical University, Shenyang 110004, China; tyxu@cmu.edu.cn (T.X.);; 2 Department of Pharmacy, Tianjin Baodi Hospital, Tianjin 301800, China; 3 Department of Pharmacy, Second Affiliated Hospital, Zhejiang University School of Medicine, Hangzhou 310009, China

**Keywords:** vancomycin, intensive care units, population pharmacokinetic, external validation

## Abstract

**Objective**: Several population pharmacokinetic (PopPK) studies of vancomycin in intensive care unit (ICU) patients have been reported. However, substantial heterogeneity exists across these studies, and their predictive performance remains uncertain owing to a lack of external validation. This study systematically reviews published PopPK models of vancomycin in critically ill patients and externally validated selected models using real-world data, with the aim of clarifying factors contributing to variability in vancomycin pharmacokinetic parameters and identifying knowledge gaps that require further investigation. **Methods**: Databases such as PubMed, EMBASE, and the Cochrane Library were searched for studies on vancomycin PopPK models in critically ill patients constructed using nonlinear mixed-effects methods. External validation of selected models was performed using the NONMEM software with external datasets. **Results**: A total of 16 PopPK studies of vancomycin in ICU patients were included. Factors such as renal function indicators, concomitant renal replacement therapy (RRT), and demographic characteristics significantly influenced vancomycin PopPK parameters in ICU patients. Serum creatinine levels and concomitant RRT significantly affected vancomycin clearance, with clearance increasing with higher creatinine clearance or the presence of RRT, whereas body weight significantly influenced the apparent volume of distribution, which increased with increasing body weight. External validation was performed for three of these models, but all models showed unsatisfactory external validation results, with poor predictive capability for the external validation datasets. **Conclusions**: The reported studies show variations in vancomycin PopPK parameters among ICU patients, with significant inter-individual variability. Clinical dosing should be adjusted based on patients’ concomitant therapies, renal function, and body weight. Existing published models lack external validation, and the results of external validation have not been satisfactory. It is recommended that PopPK models undergo external validation to assess their predictive performance before application. Additionally, future research should place greater emphasis on external validation to thoroughly evaluate model performance.

## 1. Introduction

Vancomycin (VCM) is a glycopeptide antibiotic, serving as a first-line treatment for multidrug-resistant Gram-positive bacteria such as methicillin-resistant *Staphylococcus aureus* (MRSA), and is commonly indicated for sepsis, complicated infections, and septic shock [1]. The antibacterial mechanism of vancomycin primarily involves inhibition of bacterial cell wall biosynthesis. Vancomycin binds to the disaccharide-pentapeptide precursor of peptidoglycan, specifically the D-alanyl-D-alanine terminus, on the outer surface of the cell wall, thereby preventing polymerization and cross-linking, ultimately disrupting peptidoglycan synthesis [2]. Although the use of vancomycin has increased substantially since the early 1980s owing to the widespread emergence of resistant pathogens such as MRSA, its clinical application is limited by significant interindividual pharmacokinetic variability and a narrow therapeutic window. Subtherapeutic exposure may lead to treatment failure and bacterial resistance, whereas supratherapeutic exposure may increase the risk of nephrotoxicity and ototoxicity [3]. Several international guidelines and consensuses statements recommend therapeutic drug monitoring (TDM) for vancomycin to optimize therapeutic outcomes and minimize the risk of adverse reactions [4,5,6,7,8].

In intensive care units (ICUs), patients, due to prolonged hospital stays and increased risk of nosocomial infections, necessitate frequent administration of vancomycin [9]. In critically ill patients, complex pathophysiological disturbances—including hemodynamic instability, multiple organ dysfunction, and systemic inflammatory response—together with intensive care interventions such as renal replacement therapy (RRT) and mechanical ventilation, result in substantially greater interindividual pharmacokinetic variability of vancomycin compared with that in the general population. Therefore, routine TDM is recommended for this patient population [10,11,12,13]. However, conventional trough concentration-based TDM has inherent limitations. It provides only a snapshot of drug exposure and may not fully capture the impact of dynamic changes in clinical conditions, such as fluctuating renal function or fluid shifts, on vancomycin pharmacokinetics [14]. Moreover, accurate AUC estimation often requires multiple blood samples, which may increase patient burden and, particularly in pediatric populations, potentially affect compliance [15,16]. In addition, dose adjustments are often delayed. These shortcomings are particularly pronounced in patients with rapidly changing pharmacokinetics, where TDM alone is insufficient to meet the demands of individualized vancomycin dosing.

Population pharmacokinetics (PopPK), combining traditional pharmacokinetic models with mathematical statistical models, quantitatively investigate the sources of pharmacokinetic variability and determinants of drug concentrations within a population [17,18,19]. Compared to traditional TDM, utilizing PopPK methods to construct dosing models based on maximum a posteriori Bayesian feedback (MAPB) estimation, considering the physiological and pathological states of ICU patients, is crucial for precise treatment of vancomycin in the ICU setting [20,21,22].

All published PopPK models should undergo rigorous model validation. PopPK model validation can be classified into internal and external validation based on whether the validation data is independent of the modeling data. External validation, which uses patient data different from that used in the model development, can reflect the success of extrapolative predictions of the PopPK model. This method is crucial for assessing the robustness and applicability of PopPK models [23,24,25]. However, due to difficulties in obtaining patient data, most PopPK models lack evaluation through external data validation. Differences across clinical settings may limit the generalizability of published models, and the predictive performance of these models when extrapolated to other centers remains uncertain, warranting a comprehensive evaluation of their cross-center predictive performance using external datasets.

Several studies on vancomycin PopPK models for ICU patients have been reported, but discrepancies exist across these studies, and most models lack external validation. The pharmacokinetic parameters reported across various studies urgently need to be integrated, summarized, and subjected to external evaluation. In the present study, we analyzed the covariates, final models, and pharmacokinetic parameters from published PopPK studies of vancomycin in ICU patients and externally evaluated selected models that had not been previously validated using real-world data and modeling software. This study aims to identify factors contributing to variability in vancomycin pharmacokinetic parameters, assess the predictive performance of the models, and provide a foundation for individualized treatment in ICU patients receiving vancomycin.

## 2. Methods

### 2.1. Data Source and Search Strategy

In accordance with the Preferred Reporting Items for Systematic Reviews and Meta-Analyses (PRISMA) 2020 statement [26], a systematic search of PubMed, EMBASE, and the Cochrane Library was conducted for published studies on vancomycin PopPK models in adult ICU patients, covering the period from database inception to March 31, 2026. The completed PRISMA checklist is provided in Supplementary File S1. Search terms included (“vancomycin” OR “VAN”) AND (“population pharmacokinetic*” OR “pharmacokinetic model*” OR “nonlinear mixed effect*” OR “NONMEM” OR “NLME” OR “WINNONMIX” OR “nlmixed” OR “MONOLIX” OR “Pmetrics” OR “ADAPT” OR “P-PHARM”) AND (“Critically ill*” OR “ICU” OR “Critical care*”). The detailed search strategies are provided in Table S1 of Supplementary File S2. In addition, the reference lists of all relevant articles were manually screened to ensure that no eligible studies were overlooked.

### 2.2. Inclusion and Exclusion Criteria

#### 2.2.1. Inclusion Criteria

(1) Study Population: Adult critically ill patients (>18 years old);
(2) Study Drug: Vancomycin for monotherapy or combination therapy;
(3) Study Type: Retrospective or prospective vancomycin PopPK studies;
(4) Study Method: Utilization of nonlinear mixed-effects models with complete information on final model parameters.

#### 2.2.2. Exclusion Criteria

(1) Studies conducted in vitro or in animals;
(2) Studies validating or applying previously published models;
(3) Studies not utilizing nonlinear mixed-effects models;
(4) Review articles such as systematic reviews or meta-analyses;
(5) Articles published in non-English languages.

### 2.3. Data Extraction

The title, abstract, and complete text of each article were examined by two independent investigators, who then resolved any discrepancies with a third senior investigator and reached a consensus to extract the following data. Basic Study Information: Study center, study type, number of patients, number of blood samples, etc. Patient Population Information: Patient age, gender, weight, etc. Administration Regimen and Blood Sample Collection Information: Daily dose, duration of administration, concomitant medications, concentration measurement methods, etc. PopPK Model-related Information: Analysis software, model form, inter-individual and residual variability, selection of covariates for investigation, covariate analysis methods, final model and included covariates, model validation methods, etc.

### 2.4. Quality Assessment of Included Studies

A checklist comprising 31 items was developed based on PopPK reporting guidelines proposed by Jamsen et al. [27] and clinical pharmacokinetic study reporting guidelines by Kanji et al. [28]. These items were categorized into five sections: title and abstract, study background and relevant introduction, study methods, results, and discussion or conclusion. Each item in the included study reports was matched against the checklist, and one point was assigned for each item that was met; otherwise, zero points were assigned. Compliance was used to evaluate the quality of each identified PopPK study. The compliance rate was calculated as follows:

*Compliance rate* (%) = (sum of items reported/sum of all items) × 100%

### 2.5. External Validation of Some Models with External Data

To evaluate the predictive performance of published models, an external validation was conducted on select models based on retrievable cases and the collected characteristics of model compartment structures and covariates. External validation was performed on a subset of published one-compartment models based on the feasibility of implementing the full parameter sets (including THETA, OMEGA, and SIGMA) and the availability of required covariates in our external dataset. Models with two- or three-compartment structures were not prioritized, as their validation would require richer sampling schemes than those available in our retrospective dataset to reliably inform the Bayesian prior; nevertheless, all original population parameters (fixed and random effects) were fixed to the published estimates during external evaluation without re-estimation. The selection of models for external validation was primarily driven by the completeness of covariate information in our external dataset. Specifically, we included only those models whose required covariates—such as RRT status, mechanical ventilation, body weight, and renal function indicators—were fully and reliably recorded in our retrospective cohort. Models that required additional parameters not available in our dataset (e.g., RRT intensity metrics such as effluent flow rate or blood flow rate) were excluded from validation, as their reliable evaluation was not feasible.

#### 2.5.1. External Evaluation Cohort

Retrospective data were collected from patients who received vancomycin treatment in the ICU of Shengjing Hospital of China Medical University from 1 January 2017 to 31 March 2024. This study has been approved by the Ethics Committee of Shengjing Hospital, China Medical University (Approval No. 2024PS1364K). All patients were required to have undergone at least one TDM during vancomycin treatment, with the first measurement taken at least 48 h after the initial administration. Key medication information and laboratory data were required to be complete for all included patients. Those with missing or incomplete data were excluded. Information collected included demographic data, vancomycin dosing information, concomitant medication, biochemical indices, and whether continuous renal replacement therapy (CRRT) or mechanical ventilation was performed. These variables were categorized as either continuous or categorical covariates.

#### 2.5.2. External Predictability Evaluation

The predictive performance of published models was evaluated using the nonlinear mixed-effects modeling software NONMEM^®^ (version 7.3.0, ICON Development Solutions, Ellicott City, MD, USA), and graphical reports were generated using R and GraphPad Prism 10. The specific evaluation methods are as follows.

Goodness-of-Fit Test: The external validation dataset was applied to the published models to predict individual vancomycin concentrations using the maximum a posteriori Bayesian (MAPB) method. Predicted concentrations were then compared to observed concentrations to evaluate model performance. The degree of fit and the correlation between predicted and observed values were evaluated using scatter plots of DV versus PRED (DV–PRED), DV versus IPRED (DV–IPRED), conditional weighted residuals versus time (CWRES–TIME), and CWRES versus PRED (CWRES–PRED).

Visual Predictive Check (VPC): The predictive performance of the published models was evaluated using Visual Predictive Check (VPC). For each model, 1000 Monte Carlo simulations were generated using the original model parameters, and the 5th, 50th, and 95th percentiles of the simulated concentrations were computed. Time after the most recent dose (TAD) was used as the independent variable to ensure that observations from different dosing intervals were aligned with respect to the same pharmacokinetic phase. The observed concentrations were then overlaid on the simulated prediction intervals to visually assess model performance.

Relative Prediction Error (PE%), Mean Prediction Error (MPE), and Mean Absolute Prediction Error (MAPE): These metrics were calculated using the following formulas to describe the model’s prediction bias and precision. The percentages of PE% falling within ±20% (F20) and ±30% (F30) were used to assess acceptability. A model is considered acceptable if MPE ≤ ±20%, MAPE ≤ 30%, F20 ≥ 30%, and F30 ≥ 50% [29].

(1) $$PE\%=\left(\frac{PREDi-OBSi}{OBSi}\right)\times 100\%$$

(2) $$MPE=\frac{1}{n}\sum_{i=1}^{n}\;\frac{PREDi-OBSi}{OBSi}\times 100\%$$

(3) $$MAPE=\frac{1}{n}\sum_{i=1}^{n}\;\left|\frac{PREDi-OBSi}{OBSi}\right|\times 100\%$$

PREDi: Predicted value; OBSi: Observed value.

Monte Carlo Simulations and Normalized Prediction Distribution Error (NPDE) Analysis: Monte Carlo simulations were performed 2000 times to assess model predictive performance using NPDE analysis. A good predictive performance model should exhibit NPDEs with a mean of 0 and variance of 1. To assess the predictive performance of each model, NPDEs were calculated and evaluated using three statistical tests: the Wilcoxon signed-rank test for mean (H_0_: mean = 0), the Fisher variance test for variance (H_0_: variance = 1), and the Shapiro–Wilk test for normality (H_0_: distribution is normal). To control the family-wise error rate associated with performing multiple tests on the same dataset, a Bonferroni correction was applied. Specifically, each individual *p*-value was multiplied by the number of tests (k = 3), and the adjusted *p*-values were compared against the nominal significance level of 0.05. A global *p*-value < 0.05 was considered indicative of significant model misspecification.

## 3. Results

### 3.1. Literature Inclusion Results

A total of 398 records were retrieved, with 145, 232, and 21 studies obtained from PubMed, EMBASE, and the Cochrane Library, respectively. After removing duplicate studies, 291 studies were subjected to initial screening based on titles and abstracts, of which 247 were excluded. The remaining 44 studies were selected as candidate studies for full-text review. Following application of the predefined inclusion and exclusion criteria, 16 studies were included for further analysis, with reasons for exclusion and the PRISMA flowchart depicted in Figure 1.

### 3.2. Reporting Quality Assessment

The results of the literature quality analysis based on the 31-item quality assessment tool are presented in Tables S2 and S3 of Supplementary File S2. Overall, the included PopPK studies demonstrated relatively high reporting compliance, with a median score of 82.3% (range: 67.7–90.3%). Most of the literature lacks a description of the route of administration in the title and abstract sections, which may be attributed to the relatively uniform route of vancomycin administration in ICU patients. Except for one study [30], all studies lack a description of methods for handling missing data in the Section 2. In addition, several studies did not report the pharmacokinetic rationale, sampling schedules, or statistical methods/software. Nevertheless, all studies adequately reported the remaining items, and the compliance rates were considered acceptable.

### 3.3. Characteristics of Included Studies

The included studies were published o between 2010 and 2023, and detailed information on their basic characteristics is provided in Table 1. The 16 studies were conducted in 13 different countries, with 3 studies [1,31,32] conducted in Australia, 2 each in China [33,34] and Spain [10,35], and the remaining studies in the United States, Chile, Ireland, Bosnia and Herzegovina, South Korea, Vietnam, Belgium, Canada, Germany, and France. In 6 studies [32,33,36,37,38,39], the subjects were ICU patients undergoing renal replacement therapy. A total of 5 studies [1,31,38,40,41] focused on septic patients, with 1 specifically targeting obese patients [40] and another targeting patients receiving CRRT (HVHF mode) [38]. Two studies [30,42] were conducted on patients receiving continuous injection, while the remaining studies did not further classify the ICU patient population. Most studies were conducted at single centers, with only two [30,32] being multicenter studies. Among them, the study by Economou et al. [32] was conducted at two centers located in Australia and Germany. Data for one study were sourced from publicly available network databases. Most studies were based on vancomycin blood concentration data in ICU patients to construct PopPK models, with only one study [1] incorporating both vancomycin blood concentrations and interstitial fluid (ISF) concentrations. The majority of studies analyzed vancomycin steady-state trough concentrations (C_min_) using a sparse sampling strategy, among which 4 studies [31,39,40,41] provided data on steady-state peak concentrations (C_max_), and another 6 studies [1,30,32,36,38,43] employed intensive sampling strategies (≥4 samples).

There were 11 prospective studies and 5 retrospective studies [10,31,33,37,39] included. The median number of patients in the model construction was 54 (range: 8–394), with only 3 studies [10,33,34] including over 100 patients, and sample sizes ranging from 54 to 1186 across all studies. Only one study [32] did not explicitly report the vancomycin dosing regimen, while all other studies administered intravenous doses ranging from 0.5 to 3 g daily, typically adjusting dosages based on C_min_. Most studies measured vancomycin C_min_ approximately 0.5–1 h before the next dose. Regarding PK/PD metrics, with the exception of one study [1] that did not explicitly report the PK/PD metric, all included studies specified the metric used to monitor clinical outcome. Of these, 8 studies used the AUC/MIC ratio, 6 studies used concentration indicators, and 1 used both.

High-performance liquid chromatography (HPLC), liquid chromatography–tandem mass spectrometry (LC-MS/MS), fluorescence polarization immunoassay (FPIA), chemiluminescent immunoassay (CLIA), and enzyme multiplied immunoassay technique (EMIT) are quantitative methods for the determination of vancomycin concentrations. Among these, fluorescence polarization immunoassay (FPIA) has been the most widely applied in reported studies.

### 3.4. PopPK Models

#### 3.4.1. Model Construction

Detailed information on vancomycin PopPK parameters for each study is presented in Table 2. The included studies mostly utilized NONMEM software for PopPK analysis, primarily employing the first-order conditional estimation algorithm (FOCE-I) for parameter estimation. Additionally, 5 studies [1,31,32,36,39] used Pmetrics software, and 1 study each used Monolix [30], Phoenix [40], and Kinetica [34] software for PopPK analysis. All studies employed intravenous dosing models to describe vancomycin pharmacokinetics, with 9 studies utilizing one-compartment models, 5 studies [30,32,36,38,42] employing two-compartment models, and 2 studies [1,43] utilizing three-compartment models. The median clearance rate (CL) of vancomycin across all constructed one-compartment models was 2.90 L/h (range: 1.66–7.23 L/h), with inter-individual variability (IIV_CL_) ranging from 9.38% to 41.72%. The median apparent volume of distribution was 62.855 L (range: 41.61–107 L), with inter-individual variability (IIV_V_) ranging from 12.4% to 80.1%. Two- and three-compartment models tended to underestimate CL compared to one-compartment models but exhibited larger IIV.

Apart from the six studies based on Pmetrics and Kinctica software, the remaining ten studies analyzed residual variability (RV). Among these, 6 studies [30,37,38,40,41,42] utilized a proportional model, 3 studies [10,35,43] employed an additive model, and 1 study [33] utilized a mixed model (combining proportional and additive models). The median proportional RV reported in the studies was 13.3% (range: 4.76–41.4%), while the median additive RV was 4.06 mg/L (range: 3.33 mg/L–4.30 mg/L).

#### 3.4.2. Covariate Analysis

All included studies conducted covariate analysis and model optimization to explore changes in vancomycin PopPK parameters in the ICU patient population, quantitatively analyzing sources of inter-individual variability (IIV) and residual variability (RV) and identifying covariates significantly affecting PopPK parameters such as clearance rate (CL) and apparent volume of distribution (V_d_). The study primarily employed a stepwise approach, including forward inclusion and backward elimination, to incorporate and exclude covariates, resulting in the final model containing covariates significantly influencing the parameters (refer to Table 2).

Among covariates significantly affecting CL, clinical indicators of renal function were the most common, with 8 studies [30,31,34,35,39,40,41,42] examining renal function clinical indicators as PopPK model covariates affecting CL values. One study [34] directly included serum creatinine levels (Cr_se_) as a covariate, while the remaining studies included creatinine clearance rate (CL_cr_) as a covariate. These models demonstrated that CL_cr_ is a key determinant of CL, with vancomycin clearance accelerating as CL_cr_ increases.

Combination therapy was another factor influencing CL parameters, with 7 studies [30,32,33,34,35,36,38] incorporating combination therapy as a CL parameter covariate. These combination therapies included renal replacement therapy (RRT) and mechanical ventilation, among others. Dialysis intensity (e.g., effluent flow rate) significantly increases CL. Higher effluent flow rates lead to a proportional increase in CL of vancomycin, directly influencing vancomycin dosing requirements. Additionally, 4 studies [10,33,34,43] explored demographic characteristics’ effects on CL, with weight and age being the main demographic characteristics included as covariates. Lin et al. [34] also considered burn injury and rescue medication as significant covariates for CL. Heffernan et al. [31] considered whether patients received treatment for more than 72 h as a significant covariate to describe the changes in CL.

Weight was the most frequently reported covariate affecting V_d_; increased body weight was consistently associated with higher V_d_ and sometimes with increased CL, reflecting a larger distribution volume and potentially enhanced renal clearance. One of the studies reported a 1% increase in V_d_ for every kilogram above the reference weight (70 kg), which was both statistically and clinically significant [35]. In addition, two studies [10,34] indicated that serum creatinine levels also significantly influenced V_d_, with one study [34] including age as a V_d_ covariate, and no other covariates were identified.

### 3.5. Model Validation

All studies evaluated models through internal validation. Diagnostic plots, bootstrap, visual predictive checks (VPC), and normalized prediction distribution errors (NPDE) were the most commonly used methods. Additionally, 4 studies [10,30,33,34] validated their research externally using independent datasets, providing comprehensive evaluations of model predictive performance and stability in conjunction with internal validation.

### 3.6. External Evaluation

In this study, three PopPK models, developed by Udy et al. [37], Kovacevic et al. [41], and Medellin et al. [35], were selected for external validation using NONMEM. These models were chosen based on the completeness of their reported parameters, including typical values, interindividual variability (IIV), and residual variability (RV), as well as their applicability to the available external dataset. The selection of models that are robust, relevant, and compatible with the available dataset ensures that external validation is both feasible and scientifically rigorous.

A comparative summary of the baseline characteristics between the external validation cohort and the development cohorts of the three selected models is provided in Table S4. This comparison highlights differences in demographic and clinical factors, such as RRT modality, which may contribute to the observed discrepancies in model performance. To ensure consistency with the original model development studies, creatinine clearance (CLcr) for the Medellín and Kovacevic models was calculated using the Cockcroft-Gault equation, as employed in the respective studies. For the Udy model, CLcr was calculated based on the formula described in the original publication.

While some other published models provide valuable insights into vancomycin pharmacokinetics, they could not be included in this validation due to specific limitations, such as the lack of comprehensive information on parameters like RRT-related treatment intensity (e.g., effluent flow rate, blood flow rate, or filter type) in the external validation dataset, precluding meaningful assessment of the predictive performance of models that explicitly included these covariates. Additionally, models without clear reporting of residual variability were excluded. Residual variability is essential for simulating prediction intervals and conducting diagnostic assessments such as visual predictive check (VPC) and normalized prediction distribution error (NPDE). The absence of this parameter impairs the ability to evaluate model accuracy and precision using external data.

#### 3.6.1. Basic Characteristics of Patients in the Validation Cohort

The external validation dataset included a total of 30 cases with 46 vancomycin plasma concentrations. Among them, 17 patients (56.66%) were male. The mean age was 59.63 ± 16.09 years, and the mean body weight was 66.77 ± 10.46 kg. The daily vancomycin dose ranged from 500 mg to 3000 mg. Vancomycin plasma concentrations were measured using the FPIA method, with a concentration range of 2.66 to 49.2 mg/L. A total of 8 patients (26.7%) underwent renal replacement therapy, and 20 patients (66.67%) required mechanical ventilation. Detailed patient information is presented in Table 3.

#### 3.6.2. Goodness-of-Fit Validation

The external validation datasets were used to fit three models separately, with scatter plots of DV–PRED and DV–IPRED shown in Figure 2. The closer the predicted concentrations are to the observed concentrations, the more the trend line coincides with the reference line, indicating better model fit and description of data trends. All three models exhibited a high degree of deviation in the population prediction trend lines, while individual prediction trend lines showed significant improvement. The external validation datasets comprised 46, 31, and 15 observations for the Medellín et al., Kovacevic et al., and Udy et al. models, respectively. The differences in sample size are attributable to the model-specific inclusion criteria (e.g., the Udy model was restricted to patients receiving CRRT).

The DV–PRED data for all three models were quite dispersed. However, the Kovacevic group showed a noticeable clustering trend, indicating relatively accurate data description, while the Udy group had the most dispersed data with numerous outliers, resulting in the poorest predictive performance.

The residual plots from the external validation of the three models showed systematic deviations, with clear trends particularly at higher vancomycin concentrations and for the model by Udy et al. These trends indicate potential model bias and underscore the need to incorporate additional covariates or refine the structural model to enhance predictive accuracy.

In summary, the GOF plots indicated that none of the three evaluated models were suitable for a priori dose optimization in the external dataset. Deviations observed in the DV–PRED plots suggest systematic bias in the model predictions, while better alignment in the DV–IPRED plots highlights the effect of Bayesian updating, which is not directly applicable to a priori dose adjustments.

#### 3.6.3. Visual Predictive Check

The VPCs for the three evaluated models, plotted against TAD, are presented in Figure 3, Figure 4 and Figure 5. For the Medellín et al. model (Figure 3), the observed concentrations generally fell within the 90% prediction intervals, although some deviation was observed at later TAD values. For the Kovacevic et al. model (Figure 4), the VPC demonstrated better alignment between observed and simulated data across the entire TAD range, with most observations falling within the prediction intervals. For the Udy et al. model (Figure 5), the observed concentrations were largely contained within the prediction intervals, though slight discrepancies were noted at the extremes of the TAD range.

#### 3.6.4. Prediction Error Evaluation

The results of the prediction error tests for each model are presented in Figure 6 and Table 4. According to the set evaluation criteria, only the Kovacevic et al. model had a Mean Prediction Error (MPE) within ±20%. All three models had a Mean Absolute Prediction Error (MAPE) greater than 30%, and none met the F20 and F30 criteria, indicating that the predictive performance of all three models did not meet the acceptance standards.

In the prediction error boxplots, the solid line within the box represents the median percentage prediction error (PE%), while the cross represents the mean PE%. The closer the median and mean are to 0, the higher the prediction accuracy of the model. A narrower box indicates higher prediction precision. Among the three models, the Kovacevic et al. and Udy et al. models had median PE% values close to 0. The Kovacevic et al. model had the narrowest box, indicating the highest prediction precision.

#### 3.6.5. Normalized Prediction Distribution Error (NPDE) Evaluation

The NPDE results for the three models are presented in Table 5. In the NPDE analysis, the means of all three models were close to 0; however, the mean of the Kovacevic et al. model did not pass the *t*-test, indicating a significant deviation from 0. Additionally, the variances for the Medellín et al. and Udy et al. models significantly deviated from 1. The Shapiro–Wilk (S-W) normality test results showed poor normality for the Medellín et al. model. The global *p*-values for all three models were less than 0.05, indicating poor goodness-of-fit for all models. The NPDE diagnostic plots are shown in Figure 7, Figure 8 and Figure 9.

## 4. Discussion

This study systematically analyzes published PopPK studies of vancomycin in ICU patients, with external validation of selected models using real-world data. The characteristics of each study are analyzed, factors influencing the pharmacokinetics of vancomycin in ICU patients are identified, and the robustness and generalizability of the validated models are critically assessed and evaluated. Current studies primarily use one-compartment models with intravenous administration to describe the pharmacokinetics of vancomycin in critically ill patients. Additionally, some studies have developed multi-compartment models for vancomycin, though no significant covariates affecting inter-compartmental exchange rates (Q) have been identified. The development of multi-compartmental models is significantly constrained by sparse sampling in critically ill populations. Sparse datasets often lack the temporal resolution required to accurately estimate intercompartmental dynamics, leading to challenges such as parameter identifiability issues, potential overfitting, and biased estimates [44,45,46]. These limitations highlight the importance of optimizing sampling strategies and model design to balance complexity and data availability.

In most studies, a one-compartment model adequately describes the pharmacokinetic (PK) profile of vancomycin. However, it is noteworthy that an increasing number of studies are now exploring the use of multi-compartment models for vancomycin modeling. Research indicates that, compared to one-compartment models, two-compartment models offer a more accurate representation of vancomycin’s distribution characteristics and allow for better estimation of PK parameters and inter-individual variability [30,32,36,38]. However, constructing two-compartment models demands higher data quality and larger sample sizes [47]. Although there are no strict sample size requirements for PopPK studies, researchers are encouraged to utilize large datasets for complex model development to optimize the description of vancomycin PK characteristics. A large dataset in the context of pharmacokinetic modeling should encompass not only a substantial number of patients to capture interindividual variability but also sufficient sampling density to accurately characterize the pharmacokinetics of the drug [48]. Sparse sampling, even in a large cohort, can limit the model’s ability to precisely estimate parameters, particularly for multi-compartment models or subpopulations with complex pharmacokinetics [49].

Moreover, the blood sampling strategy also influences the choice of model structure. For example, Kovacevi et al. [41], constrained by sparse sampling data, were required to use a one-compartment model with partially fixed parameters for their PopPK modeling. Therefore, researchers should carefully assess whether the average number of blood samples collected under their sampling strategy is sufficient to support quantitative analysis of potentially important factors and dose optimization in complex clinical scenarios. Notably, Abraham et al. [1] attempted to develop a PopPK model based on vancomycin concentrations in the interstitial fluid (ISF), but the results were not satisfactory. The limited success of this effort underscores the inherent challenges in characterizing vancomycin pharmacokinetics in ISF, including significant inter-individual variability in ISF concentrations and the lack of established clinical outcome benchmarks [50]. Consequently, further research is warranted to refine sampling methodologies, improve modeling approaches, and better characterize vancomycin distribution in the ISF, while blood concentration data should remain the primary focus for PopPK model development at this stage.

Fluorescence polarization immunoassay (FPIA) is the most commonly used method for measuring vancomycin blood concentrations. However, reports suggest that FPIA may result in a positive bias in vancomycin concentration measurements [51]. While recent technological improvements have mitigated this overestimation [52], the impact of FPIA on pharmacokinetic parameter estimation remains underexplored. Additionally, no conversion factors between vancomycin concentrations obtained by different analytical methods have been reported. Therefore, further research is necessary to determine these conversion factors, enhance the applicability of PopPK models, and thoroughly assess the influence of analytical methods on blood concentration data during external validation.

Vancomycin is primarily excreted by the kidneys, with over 80% of the drug excreted unchanged in the urine within 24 h [53]. Renal function can affect the drug’s pharmacokinetics, causing variability in PopPK parameters. Twelve studies included in this study examined the impact of serum creatinine or creatinine clearance on PopPK parameters. The results show that vancomycin CL significantly decreases in patients with AKI, leading to drug accumulation and increased blood concentration. Our study indicates that renal function and RRT treatment are the primary factors influencing vancomycin clearance (CL). Therefore, it is recommended to reduce the dosage in patients with impaired renal function to avoid adverse reactions. The concept of augmented renal clearance (ARC) has been increasingly discussed in critical care due to its implications for drug dosing [54,55]. ARC, characterized by a creatinine clearance (CLcr) of ≥130 mL/min/1.73 m^2^, is often observed in critically ill patients as a result of systemic inflammatory response, which increases cardiac output and renal blood flow [54]. This condition, further exacerbated by aggressive fluid resuscitation and the use of vasopressors, enhances glomerular filtration, leading to decreased serum creatinine levels, increased vancomycin elimination, and a higher risk of subtherapeutic drug exposure [56,57]. These factors highlight the importance of therapeutic drug monitoring (TDM) and dose adjustments in critically ill patients with ARC to ensure effective drug therapy.

Revilla et al. [10] demonstrate that elevated serum creatinine (SCr) correlates with an increased volume of distribution (Vd), consistent with the fluid retention observed in patients with renal impairment and the hydrophilic properties of vancomycin. However, this observation contrasts with findings from Lin et al. [34], which may be attributed to differences in patient age and lower body weight among those with renal impairment. Individualized dosing strategies should, therefore, be considered for patients with varying renal function.

RRT is commonly employed in the management of AKI [58]. Patients receiving RRT exhibit markedly altered vancomycin CL. Eight studies investigated PopPK parameter variations in patients receiving RRT, with seven studies indicating that vancomycin CL increases with RRT intensity. Our findings further suggest that the effect of RRT on CL depends not only on treatment intensity but also on the RRT modality. One study each explored the effects of three modes of continuous renal replacement therapy (CRRT)—sustained low-efficiency hemodialysis (SLED), continuous veno-venous hemodiafiltration (CVVHDF), and high-volume hemofiltration (HVHF)—as well as prolonged intermittent renal replacement therapy (PIRRT) on vancomycin clearance. The typical population values of clearance rate for those receiving HVHF and CVVHDF treatment modalities were lower compared to those receiving SLED treatment. This discrepancy may partly be explained by the approach of Kanji et al. [36], who classified SLED treatment as a categorical variable rather than incorporating RRT intensity as a continuous parameter; their study showed that receiving SLED treatment could more than double the vancomycin clearance rate. Economou et al. [32] found that patients treated with PIRRT showed a greater increase in CL compared with those receiving CRRT, suggesting that distinct vancomycin dosing regimens should be considered for different RRT modalities. Interestingly, Kirwan et al. [39] did not include CVVHDF as a significant covariate in the final model, which may be related to the inclusion of only patients receiving CVVHDF treatment for a duration greater than the instrument downtime duration. Similarly, Udy et al. [37] were unable to identify significant covariates quantifying the effect of CRRT on vancomycin PopPK parameters. Blood flow rate is a determinant of vancomycin clearance during RRT. High blood flow rates (≥240 mL/min) allow greater plasma contact with the dialyzer or filter, thereby enhancing solute removal, as demonstrated in studies employing HVHF [38]. Additionally, effluent flow rate, which includes dialysate flow and ultrafiltration rates, directly affects vancomycin clearance by promoting convective and diffusive transport [59,60]. During SLED, with an effluent flow rate of 300 mL/min, vancomycin CL was significantly higher (5.97 L/h) compared to non-SLED periods (2.40 L/h) [36]. Understanding the interplay between RRT parameters and vancomycin clearance is also essential for optimizing dosing strategies. High-intensity RRT modalities often necessitate more frequent dose adjustments to achieve therapeutic AUC/MIC targets and prevent subtherapeutic exposure. Furthermore, accurate reporting of RRT parameters, including blood flow rates, effluent flow rates, and filter characteristics, is critical for standardizing pharmacokinetic models and facilitating external validation [59]. Despite the valuable insights provided, variations in study design, patient populations, and the reporting of RRT parameters limit the direct comparisons across studies [61]. Future research should aim to standardize RRT reporting by adopting uniform definitions for blood and effluent flow rates and to explore the role of novel biomarkers, such as cystatin C, in predicting vancomycin clearance in patients receiving RRT.

Residual renal function (RRF) is a critical yet often underreported factor influencing vancomycin pharmacokinetics in critically ill patients undergoing RRT. RRF contributes substantially to vancomycin clearance, with the magnitude of its impact depending on the degree of residual kidney function and the intensity of RRT. Despite its recognized importance, RRF is inadequately captured in several PopPK models [62,63]. The three studies included highlighted the potential influence of RRF but lacked sufficient data to include it as a significant covariate [32,35,37]. Models incorporating creatinine clearance (e.g., Lin et al. [34]) partially account for RRF; however, they do not distinguish endogenous clearance from extracorporeal clearance, potentially leading to inaccurate estimation of vancomycin pharmacokinetics in patients receiving RRT. Future studies should directly quantify RRF, include it as a covariate, and consider its dynamic nature during RRT. Integrating RRF into PopPK models is essential for optimizing vancomycin dosing strategies and achieving therapeutic targets, particularly in patients with preserved kidney function or on intermittent RRT schedules.

When the clearance (CL) and apparent volume of distribution (Vd) of vancomycin administered via continuous infusion (CI) were compared with those obtained via intermittent infusion, the typical values of both parameters were found to be higher for CI. This suggests that patients receiving CI administration may require higher daily doses. Notably, studies have also indicated that CI may reduce the risk of vancomycin nephrotoxicity, but whether CI offers clinical advantages over intermittent infusion remains to be further validated [30,42,64,65].

Among the studies that investigated the influence of weight on PopPK parameters, seven studies ultimately included weight as a significant covariate. Five [1,10,35,37,41] of these indicated that weight is a covariate affecting V_d_, with greater patient weight associated with a larger V_d_. One study [33] suggested that weight is a covariate affecting CL, with increasing weight leading to higher vancomycin CL. Lin et al. [34] found that weight could simultaneously act as a significant covariate for both CL and V_d_, showing positive correlations with both parameters. Thus, adjusting dosage based on body weight is an essential strategy. Age can reflect body weight and renal function to a certain extent. Bang et al. [43] and Revilla et al. [10] indicated that vancomycin CL decreases with age. Additionally, Lin et al. [34] showed that V_d_ is smaller in elderly ICU patients. It is recommended to reduce the dosage in critically ill elderly patients and actively conduct TDM to prevent adverse reactions.

Despite the development of numerous vancomycin PopPK models, the scarcity of external validation studies significantly limits their generalizability and applicability in clinical practice. External validation using independent datasets is crucial to ensure model stability and effectiveness. In this study, three one-compartment PopPK models developed using NONMEM software were externally validated using real-world data from China. These models include the Medellín et al. model [35], designed for all ICU patients; the Udy et al. model [37], specific to patients receiving CRRT; and the Kovacevic et al. model [39], specific to patients not receiving CRRT. Among these, the Medellín et al. model demonstrated broader applicability compared with the other two. However, none of the models exhibited satisfactory predictive performance during external validation. The goodness-of-fit (GOF) plots revealed poor descriptive performance for all three models, with the Udy et al. and Medellín et al. models systematically overestimating lower concentrations, and all three models significantly underestimating higher concentrations. This suggests potential omissions of critical covariates or insufficient modeling of existing covariates during model development.

The suboptimal performance of these PopPK models in this external validation could be attributed to differences in clinical practices, assay methods, and sample handling across sites, rather than solely to the absence of specific covariates in the models. Variability in therapeutic drug monitoring (TDM) protocols, such as differences in sampling time, analytical techniques, or laboratory calibration, may introduce inconsistencies between datasets. Additionally, inter-institutional differences in patient management practices, such as RRT modalities or dosing strategies, likely contributed to discrepancies in model performance. Furthermore, all three models were developed using European cohorts, which may limit their applicability to datasets from China or other regions. Although the external validation dataset in this study was derived from a single hospital in China with a limited sample size, which constrains the robustness and generalizability of the validation, the poor external validation results still imply the complexity of applying PopPK models across different clinical settings and highlight the importance of incorporating multicenter and sufficiently powered external validation data to develop more robust and geographically diverse models, in order to minimize inconsistencies arising from clinical and methodological variability. These findings underscore the complexity of applying PopPK models across diverse clinical settings and highlight the importance of developing more robust and geographically diverse models, as well as of harmonizing external validation datasets to minimize inconsistencies arising from clinical and methodological variability.

The suboptimal predictive performance of the evaluated models may be partially attributed to the demographic and clinical differences between the external validation cohort and the original model development populations (Table S4). Notably, the external validation cohort had a higher proportion of patients receiving CRRT (26.7%) compared to the Medellín et al. (0%) and Kovacevic et al. (0%) models, which may have contributed to the substantial overestimation or underestimation of vancomycin concentrations observed in these models. Furthermore, differences in CLcr calculation methods—although we strictly adhered to the original equations—may still introduce bias due to inter-laboratory variability in serum creatinine assays and the dynamic nature of renal function in critically ill patients. The external validation cohort also exhibited a lower mean body weight compared to the Kovacevic and Udy cohorts, which could affect the volume of distribution estimates and subsequent concentration predictions. These findings underscore the importance of evaluating model performance in populations that closely match the model development cohorts, and highlight the need for model updating or recalibration when applying them to different clinical settings.

In addition, the reliance on a one-compartment structure in all three models may have further hindered their predictive accuracy. The selection of a one- or multi-compartment model is often determined by the sparsity and temporal resolution of the data. Sparse datasets frequently lack the granularity required to reliably estimate intercompartmental parameters, resulting in the adoption of simpler structural models [66,67]. While over half of the models reviewed in this study were one-compartment models due to challenges in model construction, many studies have demonstrated that two-compartment models provide a more accurate representation of vancomycin pharmacokinetics, particularly in critically ill patients with complex distribution kinetics [67]. Future PopPK research should prioritize the development of two-compartment models, employing dense sampling strategies and advanced modeling techniques to overcome data limitations and enhance predictive accuracy.

Although the population-level predictive performance of the models was unsatisfactory, individual predictions showed notable improvement across all three models. However, this does not fully validate their individual predictive accuracy, as the external dataset primarily consisted of sparsely sampled trough concentrations, limiting the robustness of individual predictions. Sparse sampling may result in apparent matches between predicted and observed values.

Based on predictive error (PE%) tests, none of the three models met the predefined acceptance criteria. The Medellín model showed a substantial positive bias with an MPE of 76.61% and MAPE of 104.82%, indicating a systematic overestimation of vancomycin concentrations. The Udy model, evaluated exclusively in the CRRT patient subset, also exhibited considerable bias (MPE: 26.91%, MAPE: 90.06%) and poor precision, with only 26.67% of predictions falling within ±30% of observed values. The Kovacevic model, evaluated in the non-CRRT subset, demonstrated the smallest bias among the three models (MPE: −12.41%), although its precision remained suboptimal (MAPE: 62.24%, F30: 29.03%). Similarly, Normalized Prediction Distribution Error (NPDE) analysis revealed substantial deficiencies in model fit. The global *p*-values for all three models were less than 0.05, indicating significant model misspecification. When considered alongside GOF plots and predictive error-based tests, the Kovacevic model showed relatively better performance within its applicable non-CRRT population, but still requires considerable refinement to meet clinical standards. It should be noted that the three models were evaluated using different subsets of the external dataset, corresponding to their original target populations. Therefore, the apparent differences in predictive performance should not be interpreted as a direct head-to-head comparison of the models themselves, but rather as an assessment of each model’s applicability to its respective patient subgroup. The overall unsatisfactory performance across all three models underscores the limited generalizability of the currently available vancomycin PPK models to our Chinese ICU population and highlights the need for model recalibration or development of new models using local data.

In summary, the reported PopPK parameters of vancomycin and their influencing factors vary across studies in ICU patients. Accounting for factors such as renal function indicators, combined RRT treatment, and body weight can help better guide individualized vancomycin therapy in this population. The results of this external validation highlight critical limitations in the robustness and generalizability of the currently established models. Discrepancies in clinical practices, methodological variability, and population heterogeneity likely account for their suboptimal performance. These findings underscore the need for future research to address these limitations by developing more robust two-compartment models, incorporating a greater number of more accurately measured covariates, harmonizing external validation datasets, and including more diverse populations, thereby enhancing the clinical utility of PopPK models.

This study has several limitations. Firstly, the literature search may have missed relevant non-English publications, as it was limited to English-language studies. Furthermore, the analysis was restricted to vancomycin PopPK models for adult ICU patients, excluding pediatric pharmacokinetics, which differ substantially from those of adults; therefore, this study does not provide insights into the pharmacokinetic parameters of the pediatric population. Finally, the external validation results reported in this study are based on a specific dataset with a sparse sampling design and a relatively limited sample size, which precluded external validation of two-compartment models. Therefore, model performance may vary across different clinical settings and patient characteristics.

## 5. Conclusions

Reported studies have shown differences in vancomycin PopPK parameters among ICU patients, with varying degrees of inter-individual variability. Clinical dosages should be adjusted based on factors such as RRT status, renal function, and body weight. Further external validation, extension, and evaluation of vancomycin PopPK models in ICU patients are necessary.

## Figures and Tables

**Figure 1:**
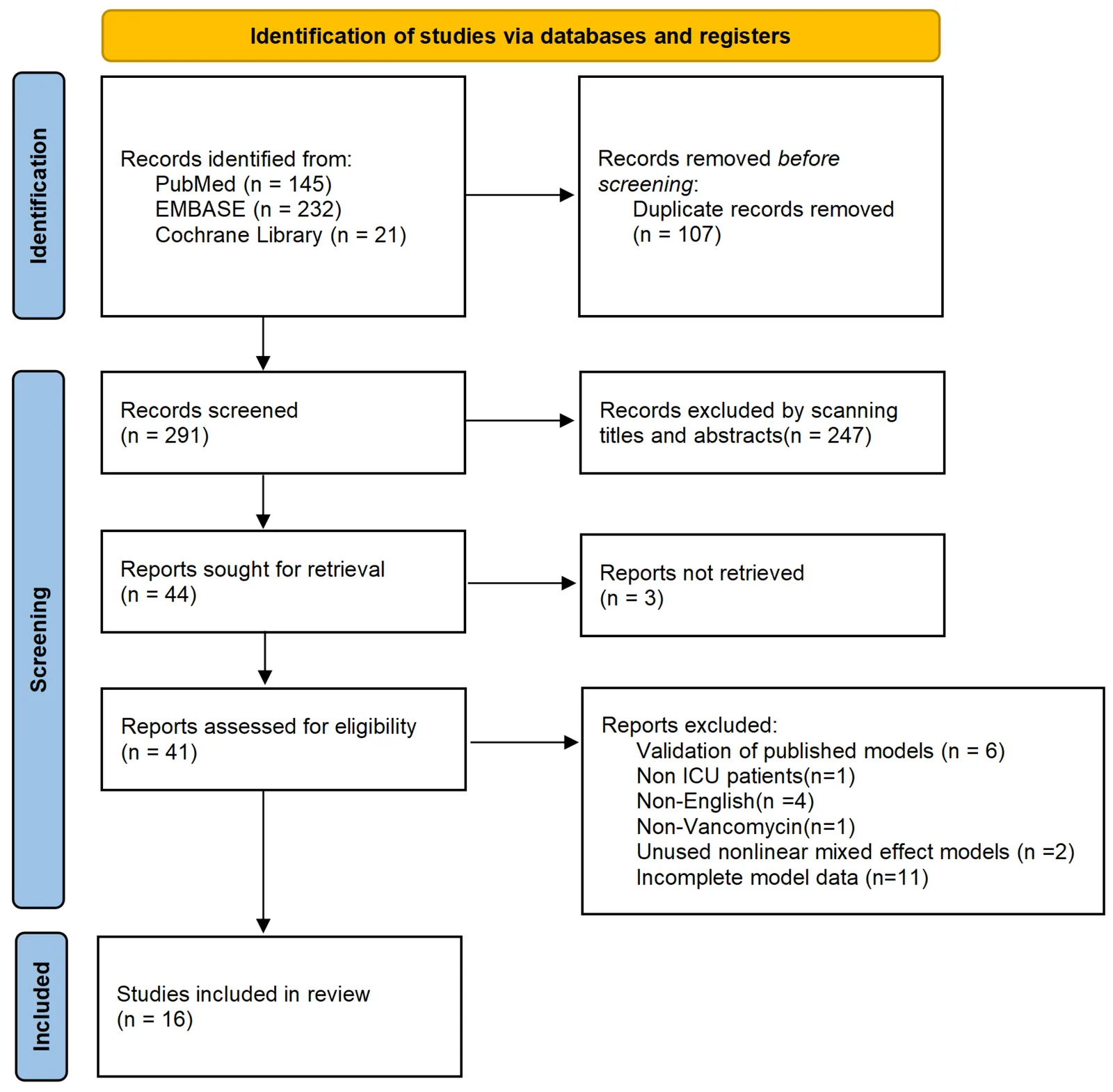
PRISMA Flowchart of Literature Retrieval.

**Figure 2:**
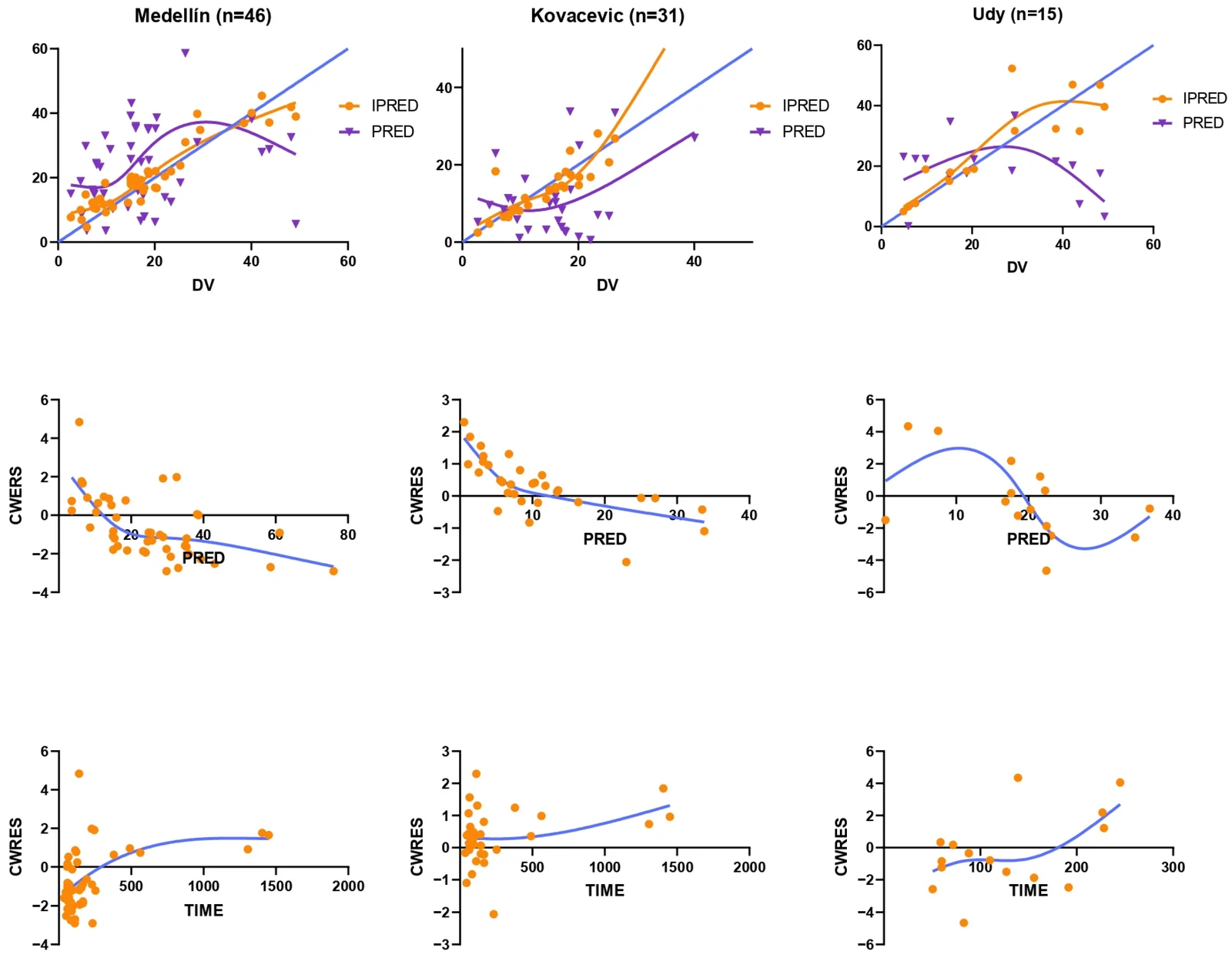
Goodness-of-fit plots.

**Figure 3:**
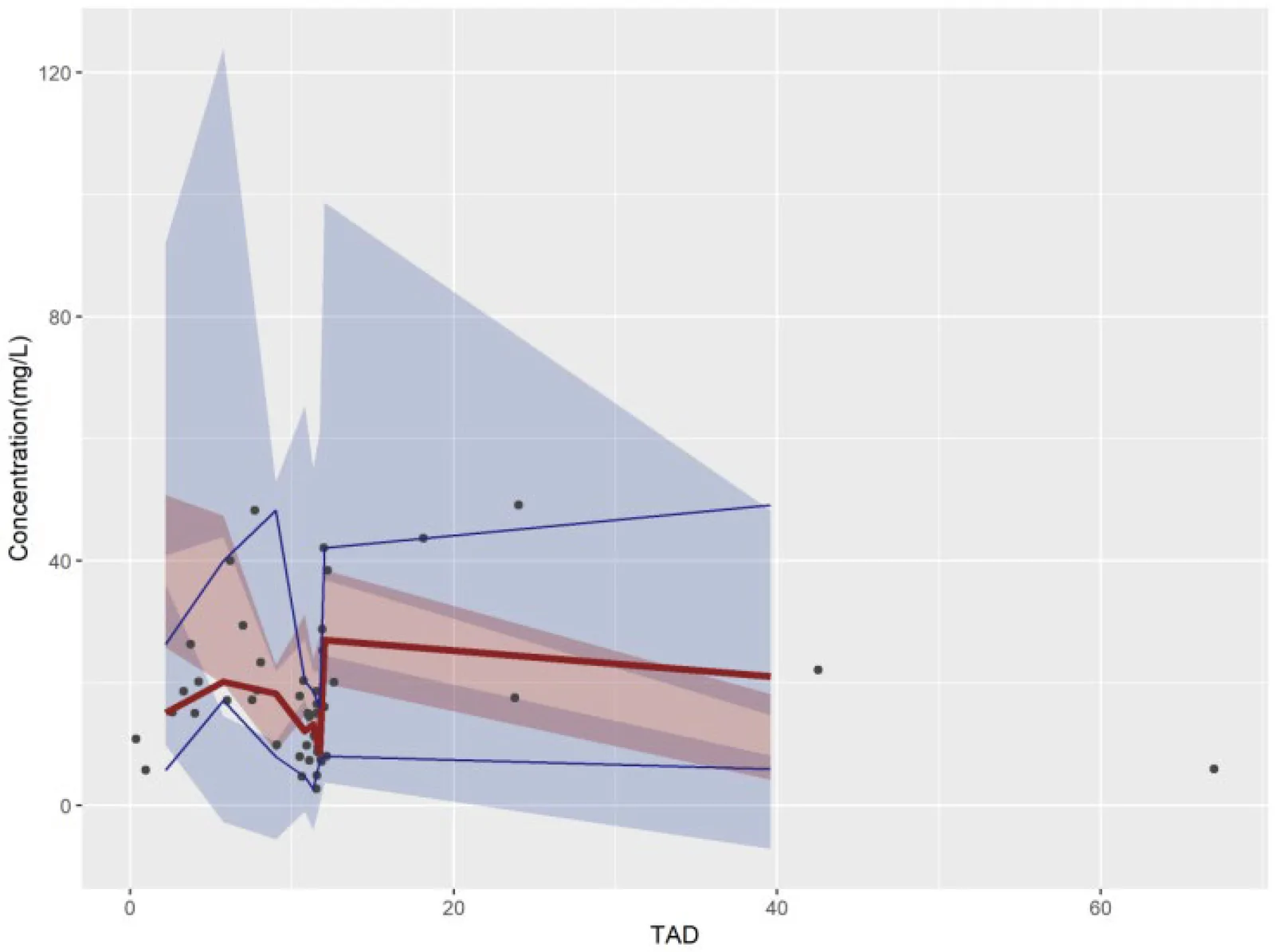
**VPC diagnostic plots for the Medellín et al. model.** The VPC was performed using 1000 Monte Carlo simulations. The red solid line represents the median of the observed concentrations, and the blue solid lines represent the 5th and 95th percentiles of the observed data, respectively. The red shaded area represents the 95% confidence interval around the median of the model-simulated predictions, while the blue shaded areas represent the 90% prediction intervals (5th to 95th percentiles) of the simulated concentrations. The independent variable is time after the most recent dose (TAD).

**Figure 4:**
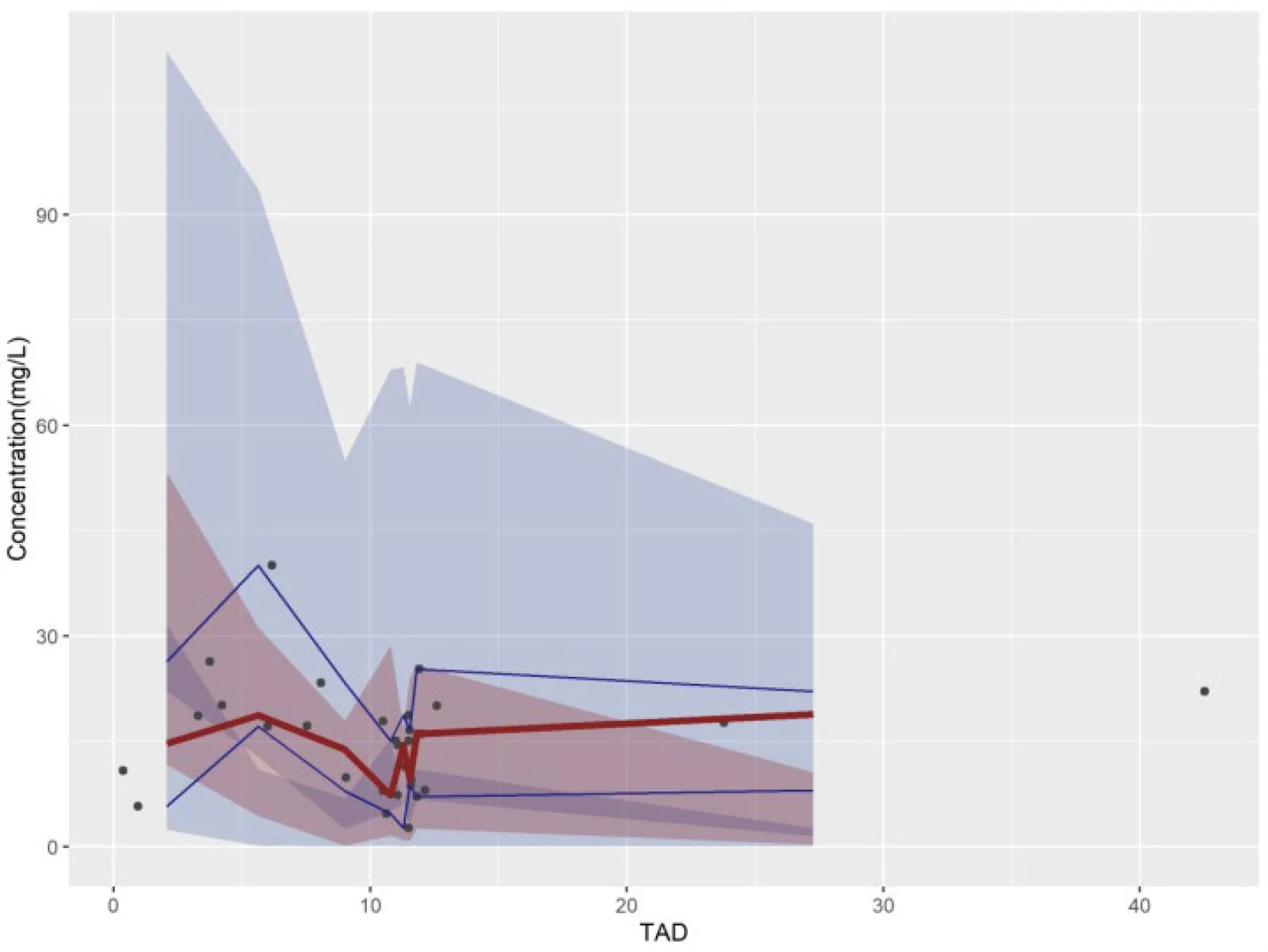
**VPC diagnostic plots for the Kovacevic et al. model.** The VPC was performed using 1000 Monte Carlo simulations. The red solid line represents the median of the observed concentrations, and the blue solid lines represent the 5th and 95th percentiles of the observed data, respectively. The red shaded area represents the 95% confidence interval around the median of the model-simulated predictions, while the blue shaded areas represent the 90% prediction intervals (5th to 95th percentiles) of the simulated concentrations. The independent variable is time after the most recent dose (TAD).

**Figure 5:**
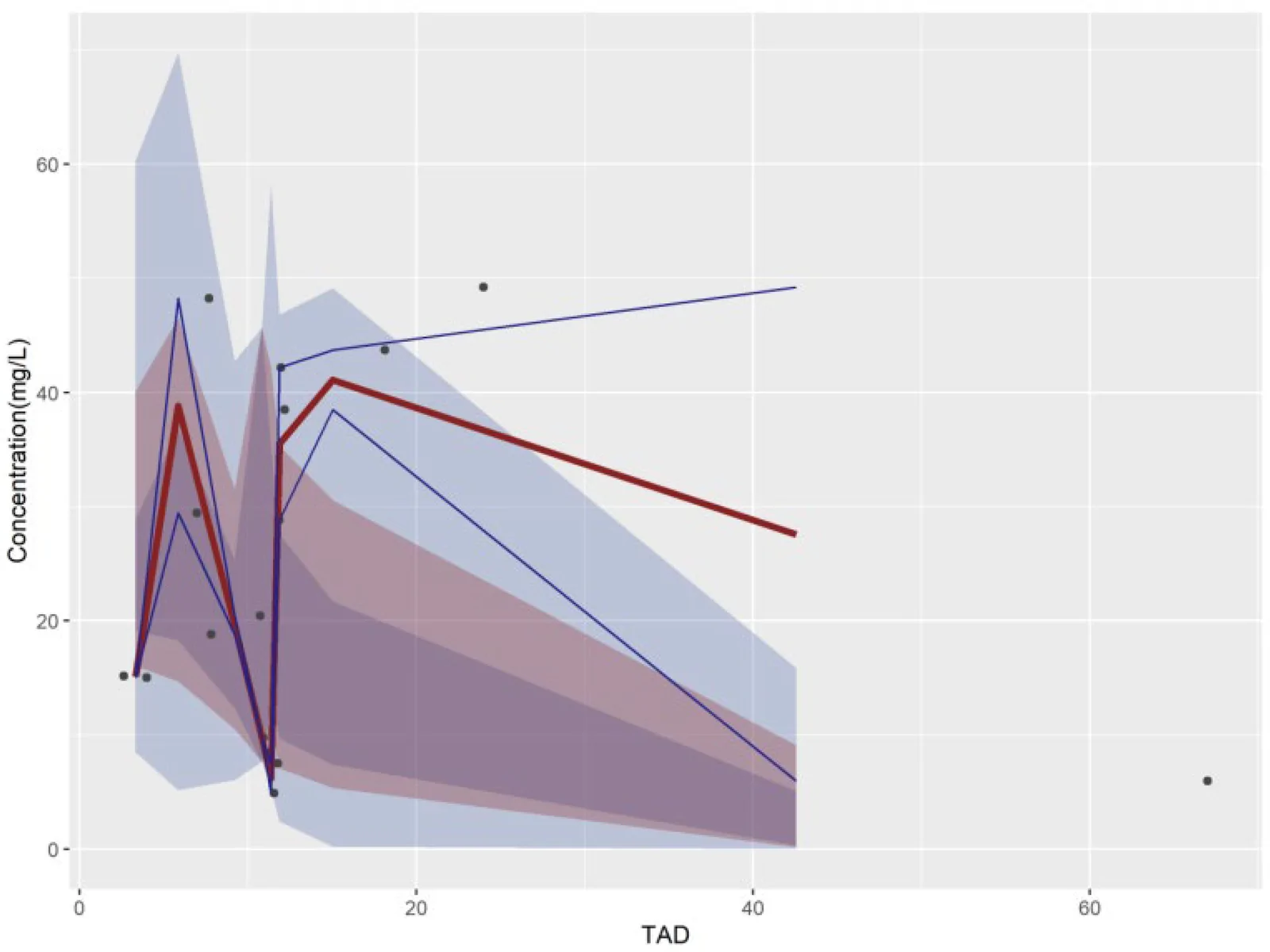
**VPC diagnostic plots for the Udy et al. model.** The VPC was performed using 1000 Monte Carlo simulations. The red solid line represents the median of the observed concentrations, and the blue solid lines represent the 5th and 95th percentiles of the observed data, respectively. The red shaded area represents the 95% confidence interval around the median of the model-simulated predictions, while the blue shaded areas represent the 90% prediction intervals (5th to 95th percentiles) of the simulated concentrations. The independent variable is time after the most recent dose (TAD).

**Figure 6:**
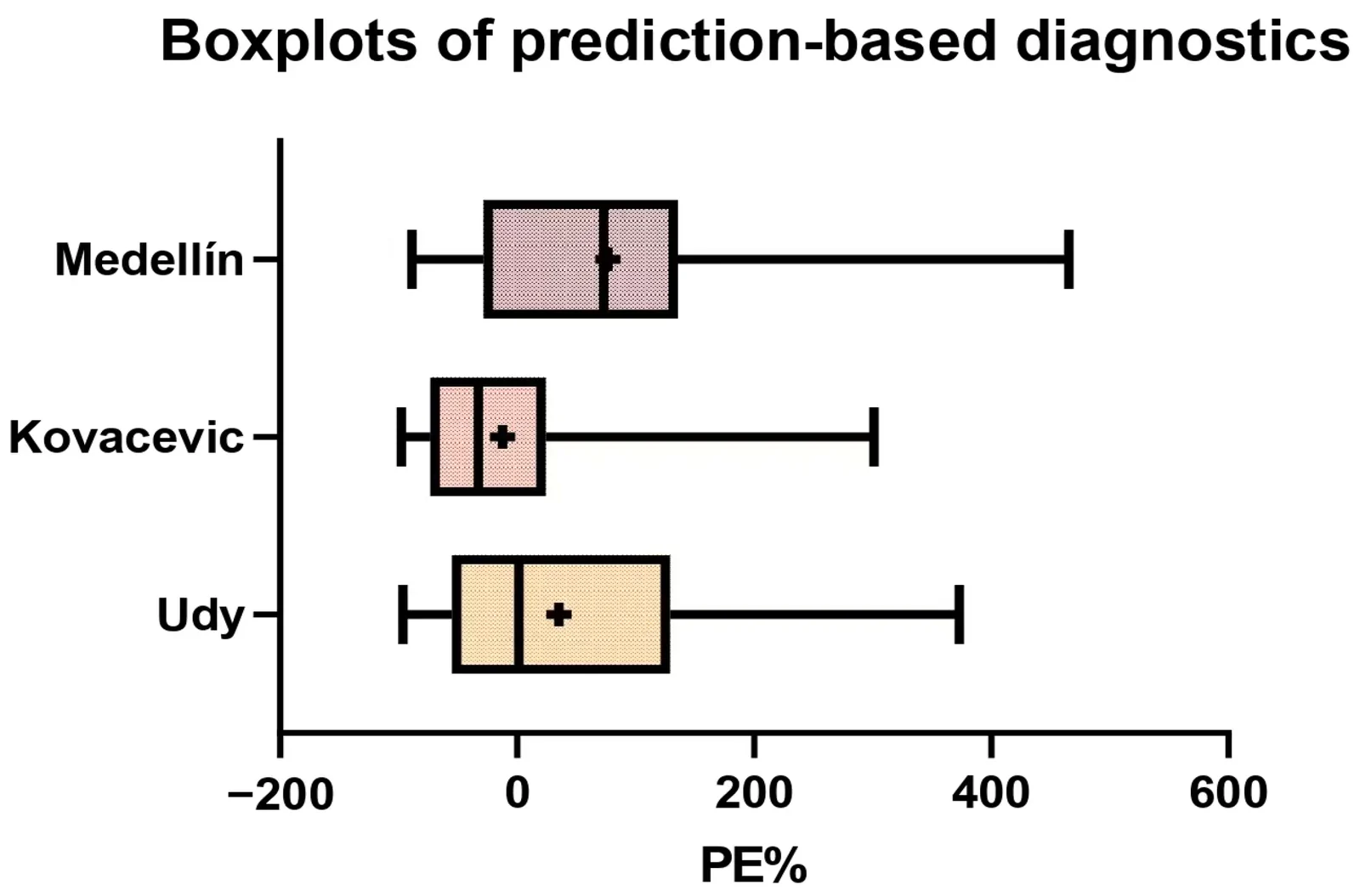
Boxplots of the percentage prediction error (PE%).

**Figure 7:**
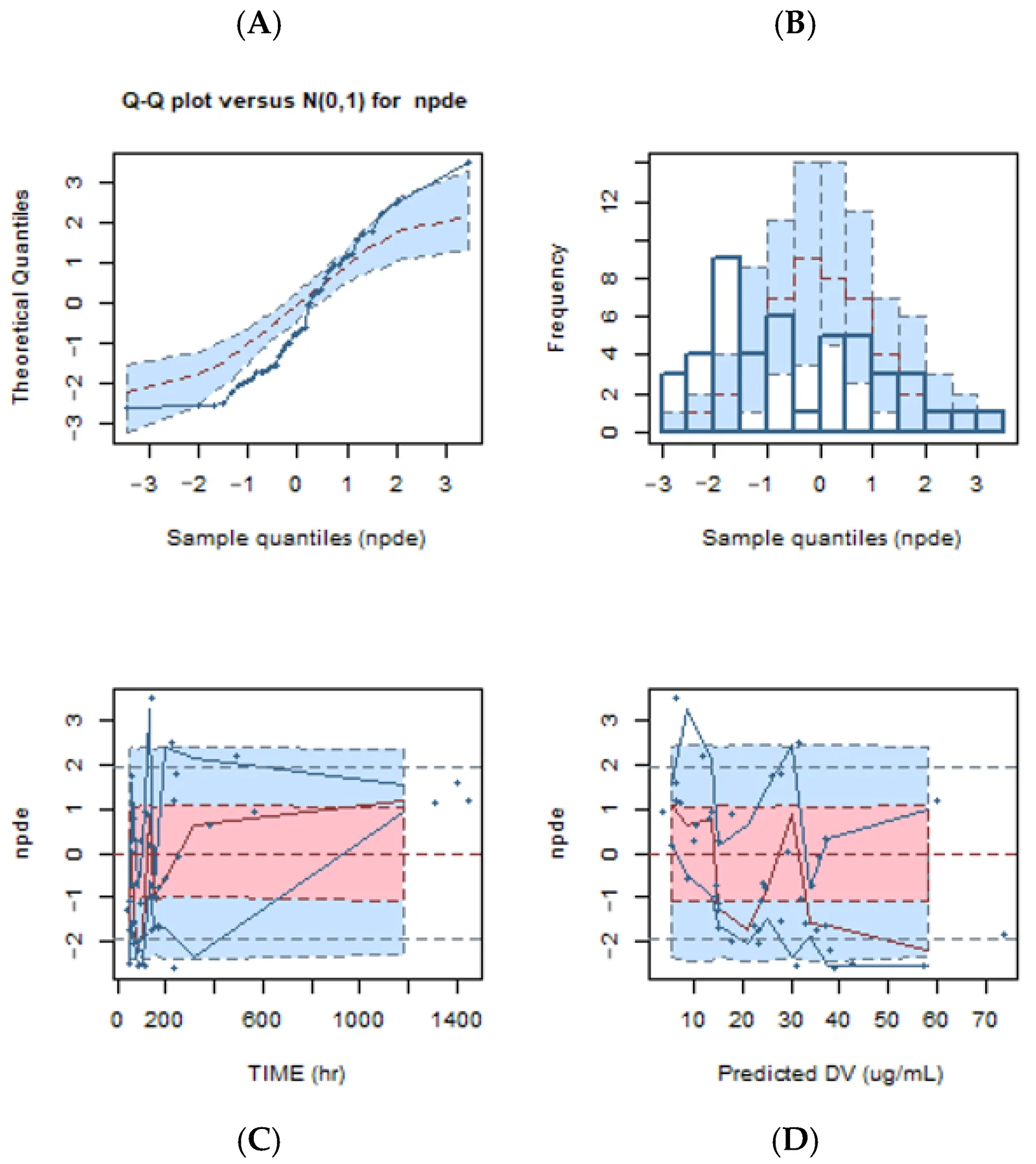
**NPDE diagnostic plots for the Medellín et al. model.** Four panels comprise the NPDE diagnostic plot set for evaluating model goodness-of-fit: (**A**) NPDE Q-Q plot: Scatter points represent the quantiles of NPDE versus the theoretical quantiles of the standard normal distribution. The solid line is the reference diagonal; close alignment of points around this line indicates that the NPDE follows a standard normal distribution. (**B**) NPDE histogram: The bars represent the frequency distribution of NPDE, overlaid with the standard normal density curve (solid line) for visual comparison of the NPDE distribution against the theoretical normal distribution. (**C**) NPDE vs. time scatter plot: The *x*-axis represents sampling time, and the *y*-axis represents NPDE values. The red solid line is the LOESS smoothing curve, and the two red dashed lines represent the mean ± 2 standard deviation boundaries, used to assess whether residuals exhibit systematic bias over time. (**D**) NPDE vs. population predictions (PRED) scatter plot: The *x*-axis represents model-predicted population concentrations, and the *y*-axis represents NPDE values. The red solid line is the LOESS smoothing curve, and the two red dashed lines represent the mean ± 2 standard deviation boundaries, used to assess whether residuals exhibit systematic bias across the range of predicted concentrations.

**Figure 8:**
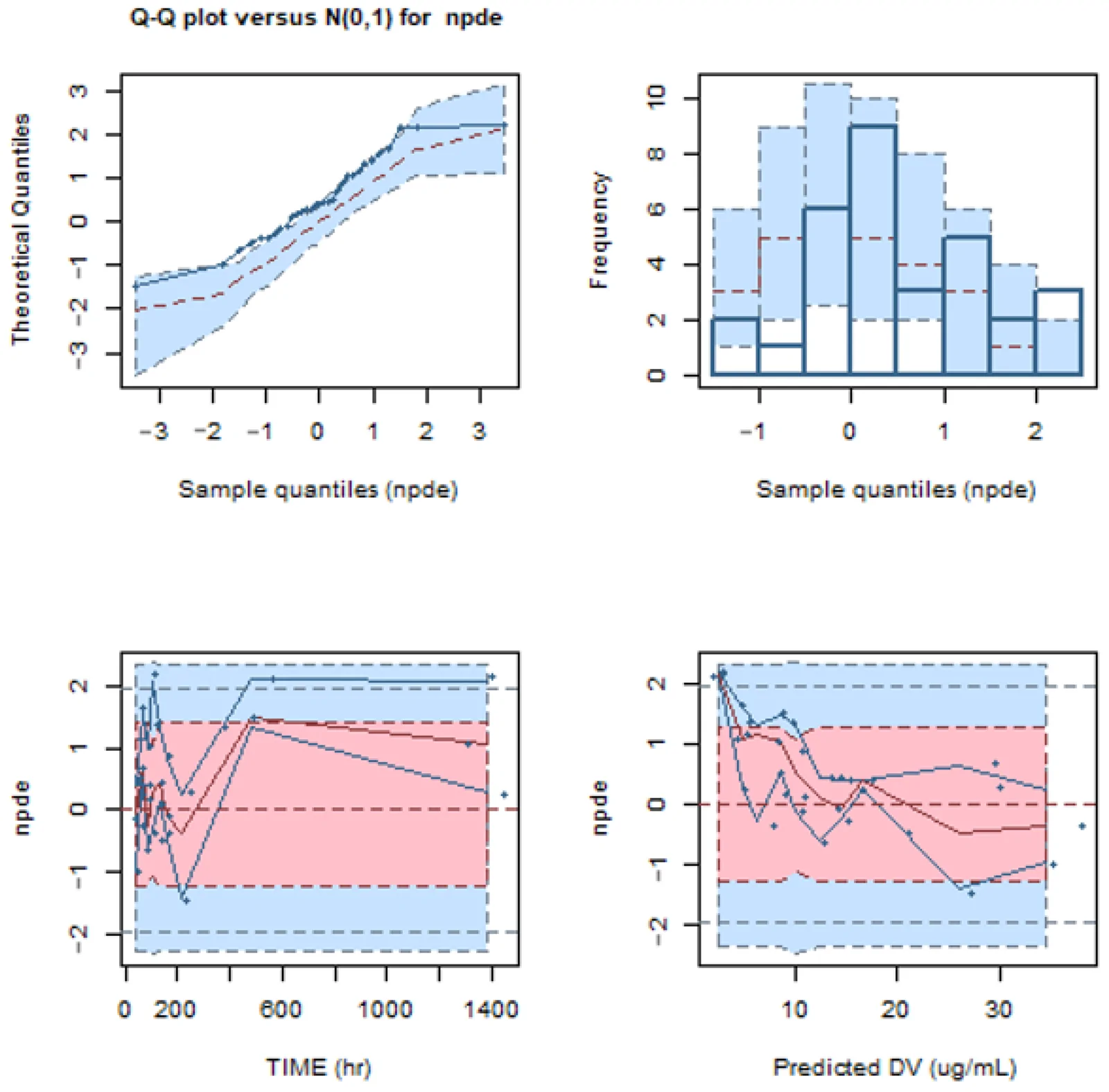
NPDE diagnostic plots for the Kovacevic et al. model.

**Figure 9:**
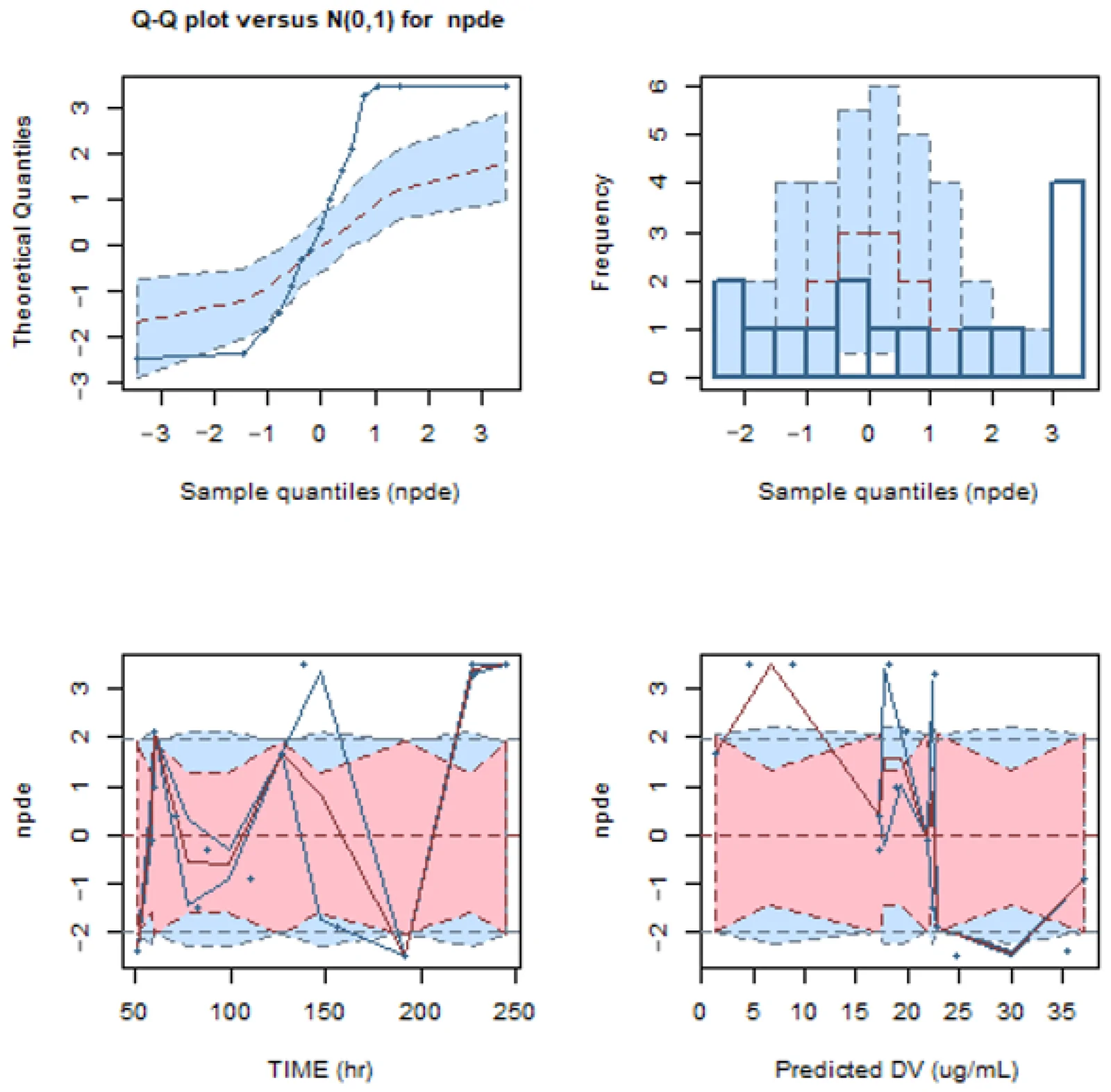
NPDE diagnostic plots for the Udy et al. model.

**Table 1:** Characteristics of population pharmacokinetic studies included in the systematic review.

Studies	Country	Type of Study	Case (*n*)	Sample (*n*)	Age (Years), Mean ± SD or Median (Range)	Dosing Regimen	Sample Type	PK/PD Metric	PD Target	Bioanalytical Method
Abraham 2019 [1]	Australia	Prospective	8	126	58 (49.7–70.0)	24.5 (19.2–26.1) mg/kg/d	C_ISF_ Intensive sampling	NR	NR	HPLC
Bang 2022 [43]	Korea	Prospective	22	197	67.5 ± 13.2	25 mg/kg	Intensive sampling	AUC/MIC	500–600	NR
Economou 2018 [32]	Australia and Germany	Prospective	11	192	53 (50–65.5)	NR	Intensive sampling	AUC/MIC	400–700	FPIA
Escobar 2014 [38]	Chile	Prospective	9	72	57 ± 14	1 g/d	Intensive sampling	C_SS_	20–30 mg/L	LC-MS
Garreau 2021 [30]	France	Prospective	78	335	68.9 ± 12.3	Initial dose: 25.2 ± 7.6 mg/kg Maintenance dose: 22 ± 5.8 mg/kg/d	C_min_	AUC/MIC	400–600	NR
Heffernan 2019 [31]	Australia	Retrospective	27	54	37 (26–49.3)	15 mg/kg	C_min_ C_max_	AUC/MIC	>451	NR
Kanji 2020 [36]	Canada	Prospective	31	335	63.0 ± 17.0	15 mg/kg/d or 1 g/d	Intensive sampling	AUC/MIC	>400	LC-MS/MS
Kirwan 2021 [39]	Ireland	Retrospective	24	106	67 (56.8–75.3)	Initial dose: 15 mg/kg Maintenance dose: 15–20 mg/kg	C_min_ C_max_	C_min_ C_max_	15–20 mg/L 20–40 mg/L	CLIA
Kovacevic 2020 [41]	Bosnia	Prospective	73	146	60 (20–87)	2 g/d	C_min_ C_max_	C_min_ AUC/MIC	12–50 mg/L >400	FPIA
Lin 2021 [34]	China	Prospective	394	837	62 (18–93)	0.5–2 g/d	C_min_	C_min_	10–20 mg/L	HPLC
Masich 2020 [40]	USA	Prospective	16	64	62 (30–78)	Initial dose: 15 (8.5–17.5) mg/kg Maintenance dose: 24 (12.6–32) mg/kg/d	C_min_ C_max_	AUC/MIC	400–600	NR
Medellín 2017 [35]	Spain	Prospective	54	641	65.0 ± 12.3	12 ± 5 mg/kg	Intensive sampling	C_SS_	20–30 mg/L	NR
Revilla 2010 [10]	Spain	Retrospective	191	569	61.1 ± 16.3	18.4 ± 10.3 mg/kg/d	C_min_	AUC/MIC	>400	FPIA
Udy 2013 [37]	Belgium	Retrospective	81	199	61.0 ± 15.6	Initial dose: 16.4 ± 5.5 mg/kg Maintenance dose: 23.7 ± 8.1 mg/kg/d	C_min_	C_SS_	20–30 mg/L	NR
Vu 2019 [42]	Vietnam	Prospective	55	247	55 ± 18	Initial dose: 19.7 ± 2.3 mg/kg Maintenance dose: 2261 ± 1052 mg/d	C_min_	C_SS_	20–30 mg/L	EMIT
Wang 2023 [33]	China	Retrospective	180	1186	64 (52–72)	Initial dose: 1000 (1000–1438) mg/d Maintenance dose: 1000 (750–1172) mg/d	C_min_	AUC/MIC	>427	NR

AUC/MIC: the area under the plasma concentration-time curve/the minimum inhibitory concentration; C_ISF_: interstitial fluid drug concentration; C_max_: peak concentration at steady state; C_min_: trough concentration at steady state; C_ss_: steady-state concentration; CLIA: chemiluminescent immunoassay; EMIT: enzyme-multiplied immunoassay technique; FPIA: fluorescence polarization immunoassay; HPLC: high-performance liquid chromatography; LC-MS/MS: liquid chromatography–tandem mass spectrometry; NR: not reported.

**Table 2:** Vancomycin Pharmacokinetic Parameter Information in ICU Patients.

Studies	Software	Model Structure	Final Model	Interindividual Variability [CV%(RSE%)]			Residual Variability	
				IIV_CL_	IIV_Vd_	IIV_Q_	Type	Value
Abraham 2019 [1]	Pmetrics	Three- compartment model	$CL(L/h)=3.33\times \frac{CL_{Cr}}{100}$ $V_{1}(L)=10.87\times {\left(\frac{BW}{80}\right)}^{0.75}$ $k_{ct}=9.17$ $k_{tc}=12.54$ $k_{pc}=4.64$ $k_{pc}=2.28$	35.64	34.42	Q_1_: 113.11 Q_2_: 47.49	NR	NR
Bang 2022 [43]	NONMEM	Three- compartment model	>65 Age: $CL(L/h)=0.858+1.626\times (\frac{IBW}{53})$ ≤65 Age: $CL(L/h)=2.094+1.626\times (\frac{IBW}{53})$ $V_{1}(L)=4.04+{\left(\frac{IBW}{59}\right)}^{4.21}$ $V_{2}(L)=16.6$ $V_{3}(L)=42.6+{\left(\frac{IBW}{59}\right)}^{12.7}$ $Q_{1}=0.294$ $Q_{2}=0.0816$	>65 Age: 21.3 ≤65 Age: NR	V_1_: 15.5 V_2_: NR V_3_: 11.1	Q_1_: 18.4 Q_2_: 33.2	Additive	3.9 mg/L
Economou 2018 [32]	Pmetrics	Two- compartment model	$CL\left(L/h\right)=3.47\times PIRRT+2.15$ $V_{c}(L)=41.85$ $k_{pc}=5.29$ $k_{CP}=5.97$	PIRRT: 57.47 NPIRRT: 96.49	58.12	K_12_: 132.97 K_21_: 125.65	NR	NR
Escobar 2014 [38]	NONMEM	Two- compartment model	$CL(L/h)=2.7+\frac{HVHF}{100}$ $V_{1}(L)=11.9$ $V_{12}(L)=17.3$ Q=4.9	NR	NR	NR	Proportional	NR
Garreau 2021 [30]	Monolix	Two- compartment model	$CL(L/h)=0.79\times e^{{\left(\frac{{CRCL}_{i}}{41.4}\right)}^{0.5}},$ if CRRT=0 $CL(L/h)=0.79\times e^{{\left(\frac{{CRRT}_{{EFR}_{i}}}{20}\right)}^{0.69}},$ if CRRT=1 $V_{1i}(L)=27.3$ $Q_{i}=6.08$ $V_{2i}(L)=61.3$	76 (11.1)	V_1_: 61(51.1) V_2_: 48(16.5)	49 (33.8)	Proportional	13%
Heffernan 2019 [31]	Pmetrics	One- compartment model	CL(L/h)<72 h $=7.23\times \frac{{CL}_{cr}}{140}$ CL(L/h)≥72 h $=5.75\times \frac{{CL}_{cr}}{140}$ V(L)<72 h=53.36 V(L)≥72 h=41.61	≥72 h: 37.52 <72 h: 35.58	≥72 h: 43.8 <72 h: 39.5	NR	NR	NR
Kanji 2020 [36]	Pmetrics	Two- compartment model	CL(L/h)=(5.97×SLED)+2.4	SLED: 67.75 NRLED: 60.64	V_1_: 78.27 V_2_: 43.34	K_12_: 104.05 K_21_: 57.61	NR	NR
Kirwan 2021 [39]	Pmetrics	One- compartment model	CL(L/h)=2.59 V(L)=80.98	18.99	20.86	NR	NR	NR
Kovacevic 2020 [41]	NONMEM	One- compartment model	CL(L/h)=0.024×CLcr+1.93 V(L)=0.511×BW	NR	NR	NR	Proportional	34.5%
Lin 2021 [34]	Kinetica	One- compartment model	CL (L/h) = 0.78 + 0.036 × DA − 0.007 × Cr_se_ − 0.43 × BurnS + 0.067 × BW + 0.41 × CRRT V(L) = 46.47 − 0.04 × Cr_se_ − 0.07 × Age + 0.16 × BW	41.72	35.14	NR	NR	NR
Masich 2020 [40]	Phoenix	One- compartment model	$CL(L/h)=3.23\times {\left(\frac{{CL}_{cr}}{40}\right)}^{0.069}$ V(L)= 85	31.5 (16.3)	12.4 (11.9)	NR	Proportional	8.89%
Medellín 2017 [35]	NONMEM	One- compartment model	Without mechanical ventilation: $CL(L/h)=2.86\times {\left(\frac{CL_{cr}}{100}\right)}^{0.75}$ With mechanical ventilation: $CL(L/h)=2.86\times {\left(\frac{CL_{cr}}{100}\right)}^{0.75}\times 0.8$ $V\left(liters/kg\right)=1.03\times BW$	28.4 (12)	49.1 (10)	NR	Additive	4.3 mg/L
Revilla 2010 [10]	NONMEM	One- compartment model	CL (L/h) = 0.67 × CLcr + Age^^(−0.24)^ ${Cr}_{se}>1\;mg/dL:$ V(L)=0.823×2.49×BW ${Cr}_{se}\leq 1\;mg/dL:$ V(L)=0.823×BW	30.13 (16)	22.83 (39)	NR	Additive	4.23 mg/L
Udy 2013 [37]	NONMEM	One- compartment model	CL(L/h)=2.9 V(L)=0.8∗BW	34.7	49.8	NR	Proportional	13.6%
Vu 2019 [42]	NONMEM	Two- compartment model	$CL(L/h)=3.63\times {\left(\frac{{CL}_{cr}}{100}\right)}^{1.01}$ $V_{1}(L)=1.01\times BW$ $V_{2}(L)=2.39\times BW$ Q=1.92	53.1 (48.9)	V_1_: 30.2 (41.2) V_2_: 62.0 (56.6)	107 (38.2)	Proportional	41.4%
Wang 2023 [33]	NONMEM	One- compartment model	CL(L/h) = 1.9 × (BW/85) × 0.797 + ITY × 0.0325 V(L)=107	9.38 (9)	25.5 (9)	NR	Mix	Prop: 4.76% Add: 3.33 mg/L

BW: body weight; CL: vancomycin clearance rate; CL_cr_: creatinine clearance rate; CRRT: continuous renal replacement therapy; Cr_se_: serum creatinine; DA: dopamine; HVHF: high-volume hemofiltration; IBW: ideal body weight; IIV: interindividual variability; ITY: CRRT intensity; K_12_, K_21_: rate constants for transfer between central compartment and peripheral compartment; kcp: rate constant for drug distribution from the central to peripheral compartment; NR: not reported; PIRRT: prolonged intermittent renal replacement therapy; Q: transfer rate constant; Q_1_: rate constant for transfer between central compartment and superficial compartment; Q_2_: rate constant for transfer between central compartment and deep compartment; SLED: subject underwent hemodialysis; V_1_: central compartment apparent volume of distribution; V_2_: peripheral compartment or superficial compartment apparent volume of distribution; V_3_: deep compartment apparent volume of distribution; V_d_: volume of distribution.

**Table 3:** Basic Characteristics of Patients in the Validation Cohort (*N* = 30).

Variable Name	Mean ± SD or *N* (Percentage; %)
No. of patients (male/female)	17/13
Age (years)	59.63 ± 16.0
BW (kg)	66.77 ± 10.46
CRRT	8 (26.7)
Mechanical ventilation	20 (66.7)
SCR (μmol/L)	67.32 ± 48.40
CL_CR_ (mL/min)	133.74 ± 89.40
ALT (U/L)	86.15 ± 178.30
AST (U/L)	123.72 ± 407.10
ALB (g/L)	28.91 ± 3.64
TP (g/L)	53.00 ± 7.68

ALB: Albumin; ALT: Alanine aminotransferase; AST: Aspartate aminotransferase; BW: Body weight; CL_CR_: Creatinine clearance; CRRT: Continuous renal replacement therapy; SCR: Serum creatinine; TP: Total protein.

**Table 4:** Prediction error assessment results.

Models	PE%		F_20_ (%)	F_30_ (%)
	MPE (%)	MAPE (%)		
Medellín et al.	76.61	104.82	10.87	15.22
Kovacevic et al.	−12.41	62.24	12.90	29.03
Udy et al.	26.91	90.06	20.00	26.67

PE%: Percentage Prediction Error; F_20_: Fraction of Prediction Error within ±20%; F_30_: Fraction of Prediction Error within ±30%; MPE: Mean Prediction Error; MAPE: Mean Absolute Prediction Error.

**Table 5:** NPDE analysis results.

Models	Mean (SD)	Variance (SD)	Skewness	Kurtosis	*t*-Test	Fisher Test	SW Test	Global *p*-Value
Medellín et al.	−0.41(0.23)	2.35(0.5)	0.049	−1.399	0.286	<0.05	0.127	<0.05
Kovacevic et al.	0.50(0.16)	0.84(0.22)	0.094	−0.443	<0.05	0.58	0.806	<0.05
Udy et al.	0.63(0.57)	4.84(1.8)	0.483	−0.645	0.080	<0.05	<0.05	<0.05

NPDE: Normalized Prediction Distribution Error; SD: Standard Deviation; SW Test: Shapiro–Wilk Normality Test.

## Data Availability

The datasets supporting the results of this study are included in the article/Supplementary Materials and are available upon request to the corresponding author for further inquiry.

## Supplementary Materials

The following supporting information can be downloaded at: https://www.mdpi.com/article/10.3390/antibiotics15100948/s1, File S1: PRISMA 2020 Checklist; File S2: Table S1: Search Strategies (Using PubMed and EMBASE as Examples); Table S2: The description of operational criteria for each of the items; Table S3: Quality assessment of studies; Table S4: Baseline Characteristics of Patients in the External Validation Cohort and the Selected Studies.
