# Supplementary material for: Systematic Review of Vancomycin Population Pharmacokinetic Models in Critically Ill Patients: A Focus on Model Characteristics and External Predictive Performance
Source: Antibiotics (Basel). 2026 Sep 24;15(10):948. doi: 10.3390/antibiotics15100948 (PMC13700732; doi:10.3390/antibiotics15100948)
Supplement: Supplementary file 1 [file antibiotics-15-00948-s001.zip › antibiotics-4480731-Supplementary File S2.pdf]

Table S1. Search Strategies (Using PubMed and EMBASE as Examples)

**PUBMED (www.ncbi.nlm.nih.gov/pudmed/advanced)**	
1	"vancomycin"[Mesh]
2	vancomycin[Title/Abstract]
3	VAN[Title/Abstract]
4	1 OR 2 OR 3
5	population pharmacokinetic*[Title/Abstract]
6	pharmacokinetic model*[Title/Abstract]
7	PPK [Title/Abstract]
8	Nonlinear mixed effect*[Title/Abstract]
9	NONMEM[Title/Abstract]
10	NLME[Title/Abstract]
11	WINNONMIX[Title/Abstract]
12	Nlmixed [Title/Abstract]
13	MONOLIX[Title/Abstract]
14	Phoenix [Title/Abstract]
15	Pmetrics[Title/Abstract]
16	ADAPT[Title/Abstract]
17	P-PHARM[Title/Abstract]
18	MWPharm [Title/Abstract]
19	5 OR 6 OR 7 OR 8 OR 9 OR 10 OR 11 OR 12 OR 13 OR 14 OR 15 OR 16 OR 17 OR 18
20	"Intensive Care Units*"[Mesh]
21	Intensive Care Units*[Title/Abstract]
22	ICU[Title/Abstract]
23	Critically ill*[Title/Abstract]
24	Critical care*[Title/Abstract]
25	20 OR 21 OR 22 OR 23 OR 24
26	4 AND 19 AND 25
**EMBASE (https://www.embase.com)**	
1	'vancomycin'/exp
2	'vancomycin':ab,ti,kw
3	'VAN':ab,ti,kw
4	1 OR 2 OR 3
5	'population pharmacokinetic*':ab,ti,kw
6	'pharmacokinetic model*':ab,ti,kw
7	'ppk':ab,ti,kw
8	'nonlinear mixed effect*':ab,ti,kw
9	'nonmem':ab,ti,kw
10	'nlme':ab,ti,kw
11	'winnonmix':ab,ti,kw

12	'nlmixed':ab,ti,kw
13	'monolix':ab,ti,kw
14	'phoenix':ab,ti,kw
15	'pmetrics':ab,ti,kw
16	'adapt':ab,ti,kw
17	'p-pharm':ab,ti,kw
18	'mwpharm':ab,ti,kw
19	5 OR 6 OR 7 OR 8 OR 9 OR 10 OR 11 OR 12 OR 13 OR 14 OR 15 OR 16 OR 17 OR 18
20	'Intensive Care Units*'/exp
21	'Intensive Care Units*':ab,ti,kw
22	'ICU':ab,ti,kw
23	'Critically ill*':ab,ti,kw
24	'Critical care*':ab,ti,kw
25	20 OR 21 OR 22 OR 23 OR 24
26	4 AND 19 AND 25
**Cochrane CENTRAL (https://www.cochranelibrary.com)**	
1	[vancomycin][Mesh]
2	(vancomycin):ti,ab,kw
3	(VAN):ti,ab,kw
4	1 OR 2 OR 3
5	(population pharmacokinetic*):ti,ab,kw
6	(pharmacokinetic model*):ti,ab,kw
7	(ppk):ti,ab,kw
8	(nonlinear mixed effect*):ti,ab,kw
9	(NONMEM):ti,ab,kw
10	(NLME):ti,ab,kw
11	(WINNONMIX):ti,ab,kw
12	(nlmixed):ti,ab,kw
13	(MONOLIX):ti,ab,kw
14	(phoenix):ti,ab,kw
15	(Pmetrics):ti,ab,kw
16	(P-PHARM):ti,ab,kw
17	(MWPharm):ti,ab,kw
18	5 OR 6 OR 7 OR 8 OR 9 OR 10 OR 11 OR 12 OR 13 OR 14 OR 15 OR 16 OR 17
19	[Intensive Care Units*][Mesh]
20	(Intensive Care Units*):ti,ab,kw
21	(ICU):ti,ab,kw
22	(Critically ill*):ti,ab,kw
23	(Critical care*):ti,ab,kw
24	19 OR 20 OR 21 OR 22 OR 23
25	4 AND 18 AND 24

Table S2: The description of operational criteria for each of the items.

Items	Suggested contents
**Title/abstract**	
The drug(s) and the patient population(s)	The title or abstract identifies the drug(s) and patient population(s) studied;
The name of the drug(s) studied	The abstract includes the name of the drug(s) studied;
The route of administration	The abstract includes the route of administration;
The population studied	The abstract includes population in whom it was studied;
Results of primary objective	The abstract includes the results of the primary objective;
Major findings	The abstract includes the major clinical pharmacokinetic findings;
**Background**	
Pharmacokinetic background	Pharmacokinetic data (i.e. absorption, distribution, metabolism, excretion) that are known and relevant to the drugs being studied are described;

Items	Suggested contents
Study rationale	An explanation of the study rationale is provided;
Specific objectives/hypotheses	Specific objectives or hypotheses are provided;
**Methods**	
Eligibility criteria	Eligibility criteria of study participants are described;
Co-administration or food	Co-administration (or lack thereof) of study drug(s) with other potentially interacting drugs or food within this study is described
Dosing	Drug preparation and administration characteristics, including dose or frequency are described;
Formulation	Drug preparation and administration characteristics, including formulation or route are described;
Sampling schedule	Body fluid or tissue sampling (timing, frequency, and storage) for quantitative drug measurement are described
Bioanalytical methods	Validation of quantitative bioanalytical methods used in the study are referenced or described if applicable
Methods for handling missing data	Methods for handling missing data such as drug concentrations below the LLOQ should also be described.
Modeling software	Pharmacokinetic modeling software used is described

Items	Suggested contents
Statistical methods and software	Statistical methods, including software used, are described
Candidate structural models	Pharmacokinetic modeling methods used is described, including assumptions made regarding the number of compartments and order of kinetics (zero, first, or mixed order)
Residual error structure	The assumptions of the distributions of the residual error structure (e.g. additive, proportional, etc.) should be described;
Covariates analysis strategy	Covariates incorporated into pharmacokinetic models are identified and described
Methods for final model evaluation	Standard goodness-of-fit plots were generated to evaluate the base model, such as observed concentrations versus population and individual predictions, histograms of subject-specific random effects, visual predictive checks [VPCs], etc.
Estimation method(s) used	Pharmacokinetic modeling estimation method used is described, such as FOCE-I.
**Results**	
Population characteristic	A table of summary clinical variables of subjects;
No. of subjects and observations	A table of summary statistics of subject demographics;

Items	Suggested contents
Table of the final model parameters	All final model parameter estimates should be listed in a table.
Final model evaluation plots	Including observed concentrations versus population and individual predictions, histograms of subject-specific random effects, visual predictive checks [VPCs], etc.
Summary of the model-building process and the derived final model	Including the results from the best base model, univariate covariate analysis, covariate selection (pivotal steps) and the final model
Plot of concentrations vs. time and/or effects vs. concentrations	A plot of drug concentrations versus time in all subjects is displayed to understand choices for candidate structural models;
**Discussion/conclusion**	
Study limitations	Study limitations describing potential sources of bias and imprecision, where relevant, should be described;
Study findings	The relevance of study findings (applicability, external validity) is described;

Table S3: Quality assessment of studies

Checklist Item	Garreau 2021	Kanji 2020	Udy 2013	Wang 2023	Escobar 2014	Masich 2020	Heffernan 2019	Medellín 2017	Lin 2021	Economou 2018	Abraham 2019	Kirwan 2021	Kovacevic 2020	Revilla 2010	Bang 2022	Vu 2019
**Title/Abstract**																
The drug(s) and the patient population(s)	+	+	+	+	+	+	+	+	+	+	+	+	+	+	+	+
The name of the drug(s) studied	+	+	+	+	+	+	+	+	+	+	+	+	+	+	+	+
The route of administration	+	-	-	-	-	-	-	-	-	-	-	-	-	-	-	-
The population studied	+	+	+	+	+	+	+	+	+	+	+	+	+	+	+	+
Results of primary objective	+	+	+	+	+	+	+	+	+	+	+	+	+	+	+	+
Major findings	+	+	+	+	+	+	+	+	+	+	+	+	+	+	+	+
**Background**																

Pharmacokinetic background	-	+	-	-	+	-	-	+	+	-	+	+	+	+	-	+
Study rationale	+	+	+	+	+	+	+	+	+	+	+	+	+	+	+	+
Specific objectives/hypotheses	+	+	+	+	+	+	+	+	+	+	+	+	+	+	+	+
Methods																
Eligibility criteria	+	+	+	+	+	+	+	+	+	+	+	+	+	+	+	+
Co-administration or food	-	+	-	+	+	-	-	+	+	+	+	-	-	+	+	+
Dosing	+	+	-	-	+	-	-	+	+	+	+	-	+	+	+	-
Formulation	-	+	-	-	-	-	+	+	+	+	+	-	+	+	+	+
Sampling schedule	+	+	-	+	+	-	-	-	+	-	+	-	+	-	+	-
Bioanalytical methods	+	+	-	+	+	-	+	+	+	+	+	+	+	+	+	+
Methods for handling missing data	-	-	-	-	-	-	-	-	-	-	-	-	-	-	-	-

Modeling software	+	+	+	+	+	+	+	+	+	+	+	+	+	+	+	+
Statistical methods and software	+	-	+	+	+	-	-	-	+	-	-	-	-	-	-	+
Candidate structural models	-	+	+	+	+	+	+	+	+	+	+	+	+	+	+	+
Residual error structure	+	+	+	+	+	+	+	+	+	+	+	+	+	+	+	+
Covariates analysis strategy	+	+	+	+	+	+	+	+	+	+	+	+	+	+	+	+
Methods for final model evaluation	+	+	+	+	+	+	+	+	+	+	+	+	+	+	+	+
Estimation method(s) used	+	+	+	+	+	+	+	+	+	+	+	+	+	+	+	+
**Results**																
Population characteristic	+	+	+	+	+	+	+	+	+	+	+	+	+	+	+	+

No. of subjects and observations	+	+	+	+	+	+	+	+	+	+	+	+	+	+	+	+
Table of the final model parameters	+	+	+	+	+	+	+	+	+	+	+	+	+	+	+	+
Final model evaluation plots	+	+	+	+	+	+	+	+	+	+	+	+	+	+	+	+
Summary of the model-building process and the derived final model	+	+	+	-	+	-	-	+	-	+	+	+	+	+	+	+
Plot of concentrations vs. time and/or effects vs. concentrations	-	+	-	+	+	+	+	-	+	+	-	-	+	+	-	-
**Discussion/conclusion**																
Study limitations	+	+	+	+	+	+	+	-	+	-	+	+	+	+	+	+

Study findings	+	+	+	+	+	+	+	+	+	+	+	+	+	+	+	+
Compliance (%)	80.6	90.3	71.0	80.6	90.3	67.7	74.2	80.6	90.3	80.6	87.1	74.2	87.1	87.1	83.9	83.9

Table S4: Baseline Characteristics of Patients in the External Validation Cohort and the Selected Studies

Characteristic	External validation cohort (n=30)	Medellín et al. 2017 (n=54)	Kovacevic et al. 2020 (n=73)	Udy et al. 2013 (n=81)
**Study country**	China	Spain	Bosnia and Herzegovina	Belgium
**Study design**	Retrospective	Prospective	Prospective	Retrospective
**Age, years**	59.6 ± 16.1	65.0 ± 12.3	60 (20–87)	61.0 ± 15.6
**Male/Female**	17 /13	NR	NR	NR
**Body weight, kg**	66.8 ± 10.5	NR	80 (50–130)	80.0 ± 18.1
**CL_{cr}, mL/min**	133.7 ± 89.4	88.6 ± 51.2	82.6 ± 41.8	NR
**CL_{cr} equation**	Cockcroft-Gault	Cockcroft-Gault	Cockcroft-Gault	NR
**CRRT, n (%)**	22 (73.3)	0 (0)	73 (73)	81 (100)
**Mechanical ventilation, n (%)**	20 (66.7)	54 (100)	NR	NR
**Dosing method**	Intermittent	Continuous infusion	Intermittent	Intermittent
**Sampling strategy**	Sparse (trough only)	Intensive (≥4 samples)	Sparse (Cmin, Cmax)	Sparse (trough)
**Sampling time points**	46 total observations	641 samples	146 samples	199 samples

CL_{cr}: creatinine clearance rate; CRRT: continuous renal replacement therapy.
